# Differential correlation analysis of glioblastoma reveals immune ceRNA interactions predictive of patient survival

**DOI:** 10.1186/s12859-017-1557-4

**Published:** 2017-02-28

**Authors:** Yu-Chiao Chiu, Li-Ju Wang, Tzu-Pin Lu, Tzu-Hung Hsiao, Eric Y. Chuang, Yidong Chen

**Affiliations:** 1 Greehey Children’s Cancer Research Institute, University of Texas Health Science Center at San Antonio, San Antonio, TX 78229 USA; 2 Graduate Institute of Biomedical Electronics and Bioinformatics, National Taiwan University, No. 1, Section 4, Roosevelt Rd., Taipei, 10617 Taiwan; 3 Department of Medical Research, Taichung Veterans General Hospital, No. 1650, Section 4, Taiwan Blvd., Xitun District, Taichung City, 40705 Taiwan; 4 Department of Public Health, National Taiwan University, Taipei, 10055 Taiwan; 5 Bioinformatics and Biostatistics Core, Center of Genomic Medicine, National Taiwan University, Taipei, 10055 Taiwan; 6 Department of Epidemiology and Biostatistics, University of Texas Health Science Center at San Antonio, 8403 Floyd Curl Dr., San Antonio, TX 78229 USA

**Keywords:** Competing endogenous RNA, Immune genes, Survival prediction, Differential correlation analysis, Glioblastoma multiforme

## Abstract

**Background:**

Recent studies illuminated a novel role of microRNA (miRNA) in the competing endogenous RNA (ceRNA) interaction: two genes (ceRNAs) can achieve coexpression by competing for a pool of common targeting miRNAs. Individual biological investigations implied ceRNA interaction performs crucial oncogenic/tumor suppressive functions in glioblastoma multiforme (GBM). Yet, a systematic analysis has not been conducted to explore the functional landscape and prognostic significance of ceRNA interaction.

**Results:**

Incorporating the knowledge that ceRNA interaction is highly condition-specific and modulated by the expressional abundance of miRNAs, we devised a ceRNA inference by differential correlation analysis to identify the miRNA-modulated ceRNA pairs. Analyzing sample-paired miRNA and gene expression profiles of GBM, our data showed that this alternative layer of gene interaction is essential in global information flow. Functional annotation analysis revealed its involvement in activated processes in brain, such as synaptic transmission, as well as critical tumor-associated functions. Notably, a systematic survival analysis suggested the strength of ceRNA-ceRNA interactions, rather than expressional abundance of individual ceRNAs, among three immune response genes (*CCL22*, *IL2RB*, and *IRF4*) is predictive of patient survival. The prognostic value was validated in two independent cohorts.

**Conclusions:**

This work addresses the lack of a comprehensive exploration into the functional and prognostic relevance of ceRNA interaction in GBM. The proposed efficient and reliable method revealed its significance in GBM-related functions and prognosis. The highlighted roles of ceRNA interaction provide a basis for further biological and clinical investigations.

**Electronic supplementary material:**

The online version of this article (doi:10.1186/s12859-017-1557-4) contains supplementary material, which is available to authorized users.

## Background

**Fig. 1:**
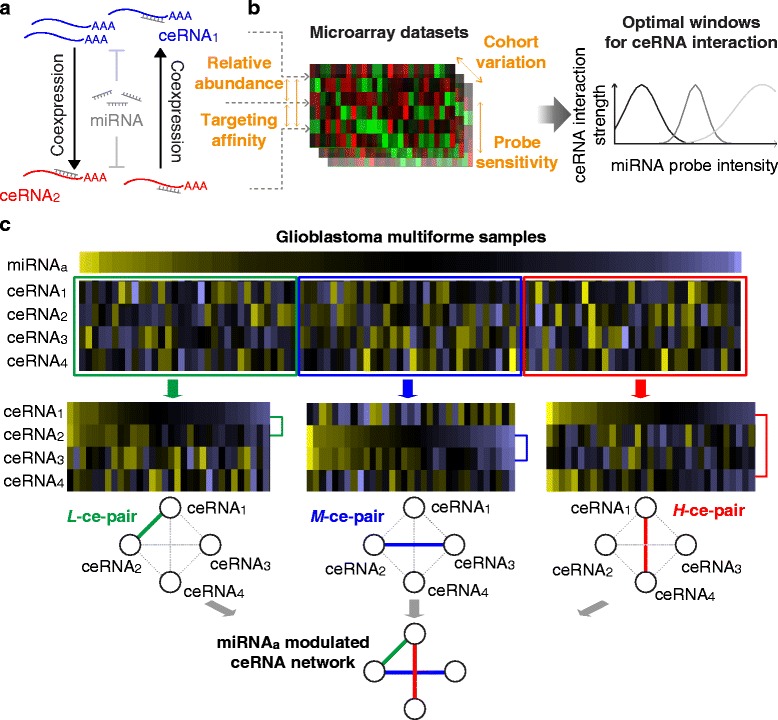
Illustration of miRNA-modulated ceRNA interaction. **a** Illustration of ceRNA interaction. An increase in the expression of one ceRNA attracts its targeting miRNAs and protects the other ceRNA from miRNA-induced degradation. Such competition leads to coexpression between two ceRNAs in a miRNA-modulated manner. **b** Based on previous reports and the characteristics of high-throughput profiling, interaction strength between two ceRNAs is optimized within a certain range of miRNA abundance. **c** Illustration of miRNA-modulated ceRNA interaction. We devised a ceRNA inference by differential correlation analysis (CEIDCA) to identify miRNA-modulated ceRNA interaction pairs. For each putative ceRNA triplet, CEIDCA partitions samples based on miRNA expression levels and statistically infers whether the two ceRNAs are differentially correlated in one group compared to another

microRNAs (miRNAs) are crucial players in tumorigenesis and patient prognosis in cancers [1, 2]. Upon complementary binding to target mRNAs, they lead to mRNA degradation or translational repression [3, 4]. Recently, an alternative role of miRNA as a “bridge” of gene interaction, namely the competing endogenous RNA (ceRNA) interaction, was proposed and verified using in vitro and in vivo models [5–7]. In the scenario of ceRNA interaction, genes (ceRNAs) achieve coexpression by competing for a common pool of targeting miRNAs (acting as sponges; illustrated in Fig. 1a). ceRNA interaction has been shown to play essential roles in the development and progression of cancers, and to potentially serve as new therapeutic targets [8–10]. Recent studies also revealed its role in propagating the effects of alterations in 3′ untranslated regions of genes, including single nucleotide polymorphisms and alternative polyadenylation [11–13]. Strength of such miRNA-mediated gene crosstalk can even outperform the one achieved by direct transcriptional regulation [14]. In vitro and in silico investigations suggest that a balance among miRNA and ceRNA abundance and targeting affinities is essential for optimal ceRNA interaction [15–19]. Taking into consideration the variations from high-throughput profiling (probe sensitivity, heterogeneity and variations of sampled cohorts, etc.), ceRNA interaction appears to be modulated by (*i.e.*, dependent on) the expression of bridging miRNAs [7, 20, 21] (illustrated in Fig. 1b). In other words, coexpression between a pair of ceRNAs (ce-pair) is optimized when its bridging miRNA is expressed within a certain range (Fig. 1b, right panel), suggesting the necessity to consider miRNA modulation in a systematic screening of ceRNA interaction.

Glioblastoma multiforme (GBM) is the most aggressive type of brain tumors. Previous works have extensively explored the molecular alterations and heterogeneity in order to better understand its biology and develop effective prognostic biomarkers [22–24]. However, 2-year and 3-year survival rates remain lower than 50% and 20%, respectively [25], suggestive of the urgent need for new therapeutic strategies. GBM was one of the cancers in which the ceRNA hypothesis was postulated and experimentally validated [6, 7], where ceRNA interaction was demonstrated to play important roles in tumor initiation and progression. Existing systematic methods for ceRNA analysis, *e.g.*, Hermes [6], are mostly built based on mutual information (MI) that requires heavy computation for statistical inference. Thus, a comprehensive investigation into the functional and prognostic significance of ceRNA interaction on a genome-wide scale that may reveal crucial biological and clinical clues to GBM remains an uncharted territory.

Addressing these needs, in the present study we devised a **ce**RNA **i**nference by **d**ifferential **c**orrelation **a**nalysis (CEIDCA) for a systematic identification of miRNA-modulated ce-pairs. Facilitated by its vastly improved computational efficiency, we conducted a genome-wide analysis and unveiled the essential involvement of ceRNA interaction in the information flow of global gene signaling, cellular signaling, and GBM-related crucial processes. Furthermore, incorporating survival data, we discovered the prognostic significance of ceRNA interactions among three immune response genes (*CCL22*, *IL2RB*, and *IRF4*), which was verified in two independent cohorts. Taken together, our data highlight the crucial roles of this alternative layer of gene interaction in GBM. The proposed method is broadly applicable to systematically explore ceRNA interaction in cancers with novel applications to regulatory biology and prognosis prediction.

## Results

### Overview of the CEIDCA algorithm

The study is aimed to comprehensively explore the functional landscape and prognostic significance of ceRNA interaction in GBM. We devised the CEIDCA algorithm to systematically identify ceRNA interaction pairs from sample-paired miRNA and gene expression profiles. Incorporating the findings from previous in vitro and in silico studies that ceRNA interaction is modulated by miRNA abundance, CEIDCA identifies a pair of ceRNAs (ce-pair) that are significantly differentially correlated with each other with changes in miRNA expression levels (Fig. 1). We defined putative ce-pair as two genes (say, *i* and *j*) that share at least one predicted targeting miRNA (*m*). For each putative ce-triplet (*i* − *j* − *m*) we tested whether the interaction strength, measured by Pearson correlation transformed to the *t*-domain (Eq. 1), between *i* and *j* changes among samples partitioned by the level of *m*. Out of simplicity, samples were stratified into high (*H*), medium (*M*), or low (*L*) expression of *m* (see the Discussion section). Statistical significance of an inter-group change (an interaction score, *ΔI*_*i*,*j*_^*m*^) was evaluated by a simulation-based lookup table. A ce-pair showing significantly intensified coexpression from one state of *m* (namely, optimized group *G* ^*opt*^) to another was identified as a miRNA-modulated ce-pair (*G* ^*opt*^-ce-pair). Details of CEIDCA are described in the Methods section (flowchart in Additional file 1: Figure S1).

### Analysis of miRNA-modulated ceRNA interaction

**Fig. 2:**
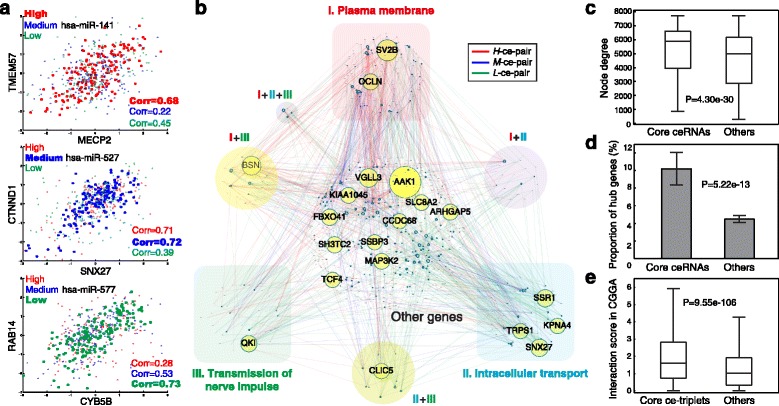
Core ceRNA interaction network and its significance in genome-wide gene interaction. **a** Examples to high- (*H*-ce-pair), medium- (*M*-ce-pair), and low- (*L*-ce-pair) miRNA-optimized ceRNA interaction pairs. Coexpression of two ceRNAs is significantly intensified in the optimized group. **b** The core ceRNA interaction network. A total of 1,762 core ceRNA triplets were identified by a stringent criterion (Bonferroni adjusted *P* < 10^-5^). Node size is proportional to node degree. ceRNAs accounting for more than 1% of edges are labeled with gene symbols. Highlighted in colors are ceRNAs involved in the largest three enriched functions reported by DAVID. **c** Node degrees of core ceRNAs and other genes in the genome-wide gene coexpression network. Statistical significance of difference is assessed by the Wilcoxon rank sum test. **d** Proportions of core ceRNAs and other genes appearing as hub genes (with degrees ranked in the top 5%) in the genome-wide interaction network. Statistical significance is assessed by a Fisher’s exact test. **e** Validation of core ceRNA triplets in CGGA datasets. Interaction scores were compared between the 1,762 core ce-triplets identified in TCGA datasets and other putative triplets by the *t*-test

In sample-paired miRNA and gene expression datasets of 520 GBM samples profiled by The Cancer Genome Atlas project (TCGA) [26, 27], we defined a total of 2,756,415 putative ce-triplets (ceRNA-miRNA-ceRNAs) that corresponded to 1,546,640 unique ce-pairs (ceRNA-ceRNAs), composed of 4,334 ceRNAs and 314 miRNAs. CEIDCA called 537,304 (3,968 ceRNAs and 304 miRNAs) significant miRNA-modulated ce-triplets (*P* < 0.01, corresponding *ΔI* >2.36). Among them, 27.0% (144,848), 21.5% (115,646), and 51.5% (276,810) were optimized in samples with high (*H*-ce-pair), medium (*M*-ce-pair), and low (*L*-ce-pair) expression of bridging miRNAs, respectively (examples in Fig. 2a). A highly interconnected ceRNA interaction network was constructed by merging these ce-pairs using Cytoscape [28] (connectivity = 270.8; Additional file 2: Figure S2A). Interestingly, the network was also highly centralized; the top 479 (12.1%) hub ceRNAs or the top 18 (5.9%) bridging miRNAs accounted for over one-half ce-triplets (Additional file 2: Figure S2B-C). The most well-studied ceRNA, *PTEN*, had an overrepresented number of ce-triplets (591, Fisher’s exact test *P* = 5.99×10^-26^). In line with findings of previous in vitro investigations (reviewed in Ref. [29]), its partner ceRNAs were associated tumor suppressive functions, such as cell death (117 ceRNAs, Fisher’s exact test *P* = 6.49×10^-12^) and apoptosis (93 ceRNAs, *P* = 2.08×10^-10^), reported by the knowledge-based Ingenuity Pathway Analysis software (IPA, Qiagen Inc.).

### Functional landscape of core ceRNA interactions

To further investigate the functional landscape of ceRNA interactions in GBM, we extracted 1,762 core ce-triplets (Fig. 2b and Additional file 3: Table S1) by a stringent criterion (Bonferroni adjusted *P* < 10^-5^, the most significant order of cutoff achievable from our simulation; corresponding *ΔI* >7.36). These ce-triplets were composed of 1,019 ceRNAs. We evaluated the role of core ceRNA interactions in global gene interaction. Notably, they had much higher node degrees in the genome-wide coexpression network than other genes (mean degrees, 5,208 vs. 4,601; Wilcoxon rank sum test *P* = 4.30×10^-30^ and *t*-test one-tailed *P* = 3.92×10^-26^; Fig. 2c), where the network was composed of gene pairs with Pearson correlation coefficient >0.099 (*P* = 0.012) that includes all core ceRNA pairs. Furthermore, a higher proportion of core ceRNAs appeared to be hub genes, defined as the top 5% genes by degree [30], in the genome-wide network (10.2% vs. 4.5%; Fisher’s exact test *P* = 5.22×10^-13^; Fig. 2d), suggesting the essential roles in maximizing information exchanges of gene interactions.

Interestingly, the top overrepresented ceRNAs, *AAK1*, *BSN*, and *SV2B* (Fisher’s exact test *P* = 1.61×10^-47^, 1.10×10^-32^, and 8.05×10^-31^; Additional file 4: Table S2), are all known to participate in cellular signaling of membrane-bound receptors or synapse [31–33]. Functional annotation analysis of the 1,019 core ceRNAs by the Database for Annotation, Visualization and Integrated Discovery (DAVID) [34] revealed concordant enrichments in synaptic/cellular signaling, accordingly plasma membrane (206 ceRNAs), cell/synapse junction (76), cytoplasmic vesicle (74), intracellular transport (108), and transmission of nerve impulse (104) (Fig. 2b and Additional file 5: Figure S3; Additional file 6: Table S3). Our findings substantiate a recent report that ceRNA is involved in routine functions of the nervous system [35], and further imply that ceRNA interaction, similar to other dynamic gene-gene interactions [36, 37], enables flexibility to the genome-wide interaction networks and facilitates cells to transiently respond to cellular stimulus and organize communication and signaling.

We also analyzed the involvement in physiology and disease by IPA. The core ceRNAs were enriched in the canonical pathway of “molecular mechanisms of cancer” (Fisher’s exact test *P* = 6.66×10^-9^) and other functions, such as “tumorigenesis of malignant tumor”, “growth of tumor”, and “epithelial-mesenchymal transition of tumor” (*P* = 5.59×10^-5^, 6.25×10^-4^, and 3.17×10^-3^).

### Validation analysis of core ceRNA interactions

We validated the core ce-triplets identified in TCGA (Fig. 2b) by an independent dataset of sample-matched miRNA and gene expression profiles (Chinese Glioma Genome Atlas (CGGA); *n* = 64) [38, 39]. We note that for cohort variability, tumor heterogeneity, and difference in profiling technologies (illustrated in Fig. 1), the validation analysis was conducted with respect to interactions scores, not examining the consistency of *G* ^*opt*^ between the two datasets. The 1,762 core ce-triplets showed concordantly increased interaction scores than other putative ce-triplets with respect to density functions (*t*-test *P* = 9.55×10^-106^; Fig. 2e) and cumulative distributions (Kolmogorov–Smirnov test statistic = 0.18, *P* = 8.78×10^-46^; Additional file 7: Figure S4). Our data suggest the reliability of CEIDCA and the stability of miRNA-modulated ceRNA interaction among cohorts.

### Comparison with MI-based methods

**Fig. 3:**
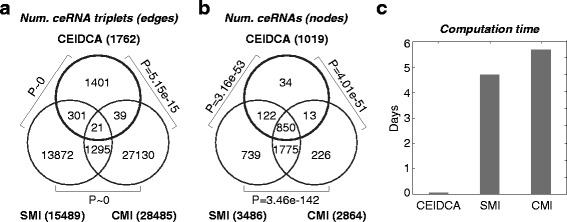
Comparison of CEIDCA to two MI-based methods. The core ceRNA network identified by CEIDCA was compared to the ones by SMI and CMI methods (*P* cutoff < 0.001) with respect to (**a**) ceRNA triplets (edges) and (**b**) ceRNAs (nodes). Significance of overlaps was assessed by Fisher’s exact tests. **c** Computation time for an inference of ~2.76 million putative ceRNA triplets

We compared CEIDCA to two MI-based methods, namely SMI and CMI (see the Methods section for details), with respect to identified ceRNA triplets (edges in the ceRNA interaction network), ceRNAs (nodes), and computation time. Since CEIDCA utilizes a random simulation in the *t*-domain, high precision of *P*-values was achieved (to the order of 10^-12^, while of 10^-3^ in SMI and CMI methods), enabling a statistically stringent inference and theoretically lowering false-positive rates. Generally, results of the three methods were highly comparable (Fig. 3a–b; Fisher’s exact test *P*-values ≤ 5.15×10^-15^ and ≤4.01×10^-51^ for identified ce-triplets and ceRNAs, respectively). While only a moderate proportion of CEIDCA-reported core ce-triplets (20.5%, 361 out of 1,762; Fig. 3a) were reported by SMI or CMI, reflecting the distinct mathematical characteristics of correlation and MI in detecting ceRNA-ceRNA interactions, we noted the two methods covered up to 96.7% of CEIDCA-identified ceRNAs (985 of 1,019; Fig. 3b). The data correspond to our previous study that suggests a massive rewiring among a stably maintained set of ceRNAs underlie ceRNA interaction networks in different cancer settings [20]. Since CEIDCA pre-generated a lookup table for the significance of interaction scores, the evaluation of ~2.76 million ce-triplets cost less than 1.4 h on a Xeon X7350 server with full 4-core 2.93 GHz processors (Fig. 3c). However, MI-based methods, for the need to permute datasets for each ceRNA triplet, are computationally expensive (4.7 and 5.7 days for SMI and CMI, respectively; Fig. 3c), limiting their applications to genome-wide analyses. More general simulation comparisons regarding MI-based vs. correlation-based methods can be found in [40].

### Prognostic significance of ceRNA interactions

**Fig. 4:**
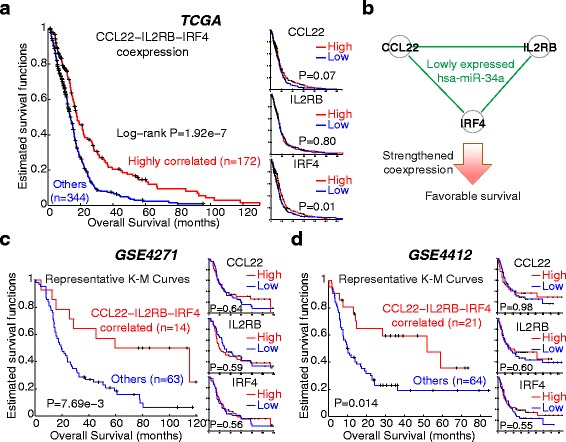
Prognostic significance of ceRNA interaction in GBM. **a** Kaplan-Meier curves of ceRNA regulatory strength, as well as expressional abundance, of three immune response genes, *CCL22*, *IL2RB*, and *IRF4*, of the TCGA dataset. **b** Illustration of the association between patients’ survival and the strength of ceRNA interaction among the three genes. In samples with lowly-expressed hsa-miR-34a, ceRNA interactions among the three genes were intensified and prognosis was improved. **c**-**d** Kaplan-Meier curves of two validation datasets. In each dataset, a machine learning procedure was performed to identify a group of samples with high average correlation among the three ceRNAs. The procedure was repeated for 1,000 iterations to ensure convergence. *Left panels*, Kaplan-Meier curves between the optimized group and others in a representative iteration (see Additional file 9: Figure S5 for summaries of all iterations). *Right panels*, Kaplan-Meier curves of individual ceRNAs

For each of the core ce-pairs, we compared the survival distributions between *G* ^*opt*^ samples and others. Interestingly, pairwise interactions among three immune response-related genes, *IRF4* − *CCL22*, *CCL22* − *IL2RB*, and *IRF4* − *IL2RB*, were found with the highest significance (log-rank test *P*-values = 1.92×10^-7^; Additional file 8: Table S4). The three *L*-ce-pairs were optimally coexpressed and associated with favorable survival in patients with low hsa-miR-34a (Fig. 4a–b). Expression levels of *CCL22* and *IRF4* were also associated with patients’ overall survival (OS) (right panel, Fig. 4a). We analyzed two mRNA-only datasets, GSE4271 (*n* = 77) [41] and GSE4412 (*n* = 85) [42], to see whether the prognostic associations were attributed to the coexpression *per se* among the three ceRNAs. Of note, expression levels of none of the three ceRNAs were significantly associated with OS (right panels of Fig. 4c–d). We designed a machine learning procedure to identify an optimized subset of samples for the three ce-pairs by iteratively including samples until the average *t*-domain correlation cannot be further increased (detailed in the Methods section). Concordantly, patients with strong positive correlation among the three ceRNAs showed significantly prolonged OS (log-rank *P* = 7.69×10^-3^ and 0.014 in the two datasets; left panels, Fig. 4c–d). To test the convergence of our machine learning procedure, we repeated the entire learning procedure for 1,000 times. Overall, patients with strong positive correlation (average transformed correlation, 12.7 vs. 3.2 and 8.9 vs. 1.1; Additional file 9: Figure S5A) showed significantly extended median OS by 9.8 (30.9 vs. 21.1) and 6.3 (18.9 vs. 12.6) months (paired *t*-test *P* = 2.70×10^-115^ and 1.24×10^-46^; Additional file 9: Figure S5B). Also, patients with strong correlation achieved higher estimated 2-year survival rates than the complete cohorts in 89.5% and 65.9% repeats. The data strongly suggest the prognostic value of modulated ceRNA interaction and warrant further investigations into *IRF4* − *CCL22* − *IL2RB* in GBM.

## Discussion

Immunotherapy is an emerging field in cancer biology and therapeutics. For the nature of the immune system to respond to cancer-specific or -associated antigens, it can be employed to attack, and even prevent, cancer cells and achieve a durable response in cancer patients [43, 44]. Regulatory T cells (Tregs) are known to infiltrate brain tumors and preferentially accumulate in high-grade ones, such as GBM [45, 46]. An investigation into GBM patients’ molecular profiles reported the association between Tregs and adverse survival [47]. Interestingly, *CCL22* (C-C motif chemokine ligand 22), one of the three ceRNAs we found to jointly predict survival, is a crucial mediator of Treg migration towards brain tumors [48–50] and specifically expressed in GBM (not in low-grade tumors) [47]. Although the other two genes in the 3-ceRNA signatures, *IRF4* and *IL2RB*, are not yet functionally characterized in GBM, they play essential roles in immune responses and cancer. *IRF4* encodes interferon regulatory factor 4, a member of the interferon regulatory factor family of transcription factors that are essential in interferon regulation in response to infection. The expression of *IRF4* is restricted to the immune system [51]. It is a critical player in the development/differentiation and the adaptive responses of B and T lymphocytes [52–55]. On the other hand, *IL2RB* (interleukin 2 receptor subunit beta) is a crucial player in T cell-mediated immune responses. It was implied to be associated with apoptosis of GBM cells [56] and to participate in IL-15 induced activation of tumor-specific gamma delta T cells [57], a subset of T cells that express a unique T cell receptor and are multi-functional in cancer [58]. Taken together with our findings, miRNA-bridged crosstalk among the 3 immune genes is implied in cell-mediated immunity. Such crosstalk may enhance cellular immune response against cancer and thus has favorable prognostic effects on GBM patients. Further investigations are necessary to delineate the underlying mechanisms.

Several interaction-based approaches to predict survival were previously developed. For instance, the dynamicity in protein-protein interactions was predictive of breast cancer outcome [36]. An association-based biomarker was proposed to distinguish patients harboring strong miRNA-gene interactions from others [59]. The biomarker was significantly associated with patient survival in both GBM and breast cancer. Recently, by incorporating a potential modulator feature, *NPM1* mutation, we proposed a prognostic predictor for acute myeloid leukemia based on the interaction strengths between several miRNAs and their modulated target genes [60]. These reports showed the value to consider, in addition to expressional abundance, the interaction strength between genomic features for the development of prognostic markers. Our discovery of the *CCL22* − *IL2RB* − *IRF4* signature illuminated the potential of ceRNA interaction as a novel candidate for the interaction-based biomarkers. Though the signature was identified by stratifying patients according to the expression levels of hsa-miR-34a in TCGA data, we note that our further analysis verified the prognostic effect of the interaction strength *per se* by using two independent mRNA-only datasets. The finding echoes a central concept of ceRNA scenario that bridging miRNAs serve as only “buffers” or “sponges” of protein-coding genes that execute biological functions.

CeRNA interaction requires the correlation between two mRNAs modulated by miRNA expression levels. To systematically analyze ceRNA interaction, Hermes [6] was developed by comparing the MI between a miRNA and one ceRNA against the conditional MI given the expression profile of another ceRNA. Although MI is a widely used measure of coexpression in genomic interaction networks [61, 62], it poses a heavy computational burden due to the permutation-based statistical inference and is limited in the application to genome-wide studies. Instead, for the mathematical transformability, correlation-based methods are computationally efficient and biologically straightforward alternatives. Studies have confirmed its efficiency and comparable, or even better, performance to MI-based methods in simulated and patients’ datasets [40, 63]. Concordantly, we showed that CEIDCA identified results highly comparable to those achieved by MI-based methods (Fig. 3a–b), while greatly improving computation efficiency and statistical stringency, identify a core set of ceRNA triplets from a genome-wide study. We also note that the 3 prognostic ce-pairs cannot be identified by the MI methods (SMI *P*-values ranged from 0.18 to 0.86; CMI *P*-values falling between 0.20 and 0.95). Incorporating findings from a synthetic gene circuit or mass-action modeling [17, 18, 21], CEIDCA was designed based on a stratification of samples according to the expression level of a bridging miRNA. Out of simplicity, we set the number of groups at three (high, medium, and low). While an increase in the number of groups (*k*) equips the method the capability to identify ceRNA pairs with subtle changes, it may compromise sensitivity to systematic noises, statistical power, and computational efficiency. To understand the effect of this parameter on CEIDCA, we compared the core ceRNA interaction pairs identified by different settings (*k*∈ [3, 10]). Notably, the results seemed to be quite stable (Fisher’s exact test *P*-values < precision of double-precision floating point, and Jaccard indices > 0.39, pairwise comparisons with the setting of *k* = 3), suggestive of the robustness and wide applicability of CEIDCA, while computation time rose roughly linearly from 1.4 to 4.7 h with *k*.

Future studies may address several limitations of this work. First, since the TCGA gene expression dataset was derived by DNA microarrays, ceRNAs analyzed in this study are mostly protein-coding mRNAs. However, some long non-coding RNAs (lncRNAs), such as a well-known oncogene, *HOTAIR*, and pseudogenes (*e.g.*, *PTENP1*), perform their functions, at least partially, by acting as ceRNA partners of crucial mRNAs [64–66]. Such lncRNA-miRNA-mRNA scenario was overlooked in this study. Next, CEIDCA analyzes each of these putative triplets independently. Realizing those interactions among miRNAs and mRNAs are highly complex in living cells, ceRNA triplets may be to some extent associated with each other and form higher-order ceRNA modules, which poses great computation burden and mathematical complexity to further investigations. Future studies may, based on our findings, conduct network-based analysis to dissect higher-order graph properties among ceRNA triplets. Furthermore, we corroborated the prognostic value of the 3-ceRNA signature by stratifying patients based on the average pairwise correlation. A per-sample prediction (*e.g.*, a prediction score) was not developed. Further studies may incorporate mathematical advances in interaction-based prediction and translate our results into a personalized biomarker. Also, though we have validated its prognostic value in two independent cohorts, analysis of other large datasets is needed before it can be applied clinically.

## Conclusions

**Fig. 5:**
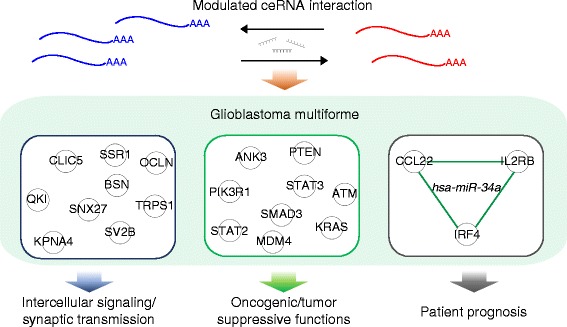
Summary of discoveries from this study. ceRNA interaction participates in crucial processes in brain, such as intercellular signaling and synaptic transmission, as well as oncogenic or tumor suppressive functions in GBM, including growth of tumor, tumorigenesis of malignant tumor, proliferation of malignant tumor, and proliferation of tumor cells. Furthermore, interaction strength among three immune response genes, *CCL22*, *IL2RB*, and *IRF4*, is predictive of patient prognosis

This work addresses the lack of a comprehensive exploration into the functional and clinical relevance of ceRNA interaction in GBM. We devised a novel and efficient algorithm that integrates miRNA and gene expression profiles of patients. As summarized in Fig. 5, by the proposed comprehensive and efficient algorithm, we showed that miRNA-modulated ceRNA interaction is involved in synaptic transmission as well as tumor-related functions in GBM. Furthermore, this is, to our knowledge, the first study to show that the interaction strength *per se* of three immune ceRNAs, *CCL22*, *IL2RB*, and *IRF4*, is predictive of patient prognosis. Overall, our findings illuminate the potential of ceRNA interaction in prognostication and therapeutics of the malignancy and warrant further biological and clinical investigations.

## Methods

### GBM datasets

We analyzed sample-paired datasets of miRNA and gene expression of 520 GBM patients profiled by TCGA [26, 27]. The two datasets were profiled by Agilent 8 × 15K Human miRNA-specific microarrays and Affymetrix HT Human Genome U133 Array Plate Sets, respectively, and we used pre-normalized level-3 data. The identified core ce-triplets were validated by independent CGGA datasets of miRNA and gene expression profiles of 64 GBMs [38, 39]. To verify the results of survival analysis, we included two additional gene expression datasets from the Gene Expression Omnibus (accession numbers, GSE4271 (*n* = 77) [41] and GSE4412 (*n* = 85) [42]). For genes with multiple probes, the one with the largest coefficient of variation was chosen as a representative probe.

### The CEIDCA algorithm

The CEIDCA algorithm was developed to systematically identify miRNA-modulated ceRNA interaction pairs. It contains three major components: i) definition of putative ce-pairs, ii) measuring miRNA-modulated interaction strengths of putative ce-pairs, and iii) statistical inference. In the first component (Additional file 1: Figure S1A), we processed TargetScan [67] prediction data for miRNA targets (downloaded from the miRSystem database [68]) as previously described [69]. A putative ce-pair was defined as two genes sharing at least one targeting miRNAs; a ce-triplet referred to the set of such ce-pair and the targeting miRNA.

For each putative ce-triplet, say ceRNAs *i* and *j* and miRNA *m*, in the second component of CEIDCA (Additional file 1: Figure S1B), samples were partitioned into *k* equally-sized groups (*G* ∈ {*G*_1_, ⋯, *G*_*k*_}) based on the expression level of *m*. We measured the interaction strength between *i* and *j* within group *G* by a Pearson correlation coefficient *ρ*_*i*,*j*,*m*_^*G*^. We eliminated non-informative ceRNAs of which coefficients of variation were less than 5% in any group [40]. Since correlation coefficients are biased by population correlation (the change of correlation coefficients from 0 to 0.2 is statistically inequivalent to one from 0.8 to 1), we transformed the coefficients to the *t*-domain [70]:

1 $$ {\boldsymbol{I}}_{\boldsymbol{i},\boldsymbol{j}}^{\boldsymbol{m}}={\left\{{t}_{i, j, m}^G\right\}}_{G\in \left\{{G}_1,\cdots, {G}_k\right\}}={\left\{{\rho}_{i, j, m}^G\sqrt{\frac{N-2}{1-{\rho_{i, j, m}^G}^2}}\right\}}_{G\in \left\{{G}_1,\cdots, {G}_k\right\}}, $$

where *N* denotes the group size. We measured the inter-group change in normalized correlation coefficients as an “interaction score” by

2 $$ \Delta {I}_{i, j}^m= max\left(\left\{{\boldsymbol{I}}_{\boldsymbol{i},\boldsymbol{j}}^{\boldsymbol{m}}\left({G}_P\right)\left|{\boldsymbol{I}}_{\boldsymbol{i},\boldsymbol{j}}^{\boldsymbol{m}}\left({G}_P\right)>0\right.\right\}\right)- min\left(\left|{\boldsymbol{I}}_{\boldsymbol{i},\boldsymbol{j}}^{\boldsymbol{m}}\right|\right), $$

and tested the following hypotheses:

3 $$ \left\{\begin{array}{c}\hfill {H}_0:\ \Delta {I}_{i, j}^m=0\hfill \\ {}\hfill {H}_1:\ \Delta {I}_{i, j}^m>0\hfill \end{array}\right.. $$

We note that since only positive interaction is considered in the ceRNA scenario, *ΔI*_*i*,*j*_^*m*^ is set as 0 if ***I***_***i***,***j***_^***m***^ has no positive element. Lastly, the significance level was assessed against an empirical density function ***D*** ^*Δ****I***^ generated by a trillion-time simulation of *t*-distributions (Additional file 1: Figure S1C):

4 $$ P\left(\Delta {I}_{i, j}^m\right)=\raisebox{1ex}{$\left|{\boldsymbol{D}}^{\Delta \boldsymbol{I}}>\Delta {I}_{i, j}^m\right|$}\!\left/ \!\raisebox{-1ex}{$\left|{\boldsymbol{D}}^{\Delta \boldsymbol{I}}\right|$}\right.. $$

We employed such a large number of simulations to enable Bonferroni correction addressing the multiple comparisons problem. The group *G* ^*opt*^ that corresponds to *max*(***I***_***i***,***j***_^***m***^) for a significant ce-pair is referred to as the “optimized group” and the ce-pair as a “*G* ^*opt*^-ce-pair”. Out of simplicity, analysis of this study was conducted under the setting of *k* = 3 (*i.e.*, *G* ∈ {*H*, *M*, *L*}, representing high-, medium-, and low-miRNA expression).

### Implementation of MI-based methods

We compared CEIDCA to two MI-based methods, SMI and CMI. SMI was implemented by simply substituting *t*-domain correlation by MI in the calculation of an interaction score, with other procedures remained identical to CEIDCA. The CMI method (slightly adapted from a previous report [6]) tests the improvement of MI between *m* and one ceRNA *i* given the other ceRNA *j*, *i.e.*, *ΔI*_*i*,*j*_^*m*^ = *CMI*(*i*, *m*|*j*) − *MI*(*i*, *m*). We used the “MIToolbox for C and MATLAB” [71] for the calculation of MI and CMI. Statistical significance was assessed by 1,000 random permutations.

### Survival analysis

To infer whether a miRNA-modulated ceRNA interaction is associated with prognosis, for each ce-pair we compared the survival curves between samples belonging to the optimized group and the others by a log-rank test. For each validation dataset, we used a machine-learning procedure to identify the optimized group of samples where a set of ceRNAs are (globally or locally) maximally correlated. We started by repeatedly randomly selecting 10% samples for 50,000 times and chose the selection achieving the largest average pairwise correlation (in the *t*-domain) as a seed. Subsequently, we iteratively added one sample into the seed that increased the average correlation by the most. The iterative procedure was terminated when the addition of any sample could not improve the correlation any more. Comparison of survival curves between the identified optimized group and the other samples, along with a comparison between samples with high and low expression of a ceRNA, directly verifies whether the predictive value of a ce-pair is confounded by the expression level *per se* of component ceRNAs. The entire machine-learning procedures were repeated for 1,000 times to ensure convergence.

## Additional files

Additional file 1: Figure S1. Flowchart of CEIDCA. The algorithm is mainly built on three components. (A) Definition of putative ceRNA triplets. We defined putative ceRNA triplets by reprocessing prediction miRNA-target data of TargetScan. (B) Measuring interaction strengths of ceRNA pairs. For each putative ceRNA triplet, GBM samples were sorted and divided into three equally-sized groups based on the expression of miRNA. We employed Pearson correlation coefficients and conversion to the *t*-domain to measure the interaction strength between two ceRNAs. (C) Statistical inference of miRNA-modulated ceRNA pairs. We tested whether a putative ceRNA pair exhibited intensified correlation in one group compared to another. The statistical significance was assessed against a trillion-time simulation. (PDF 367 kb)
Additional file 2: Figure S2. Complete ceRNA interaction network. (A) ceRNA interaction network constructed by merging 537,304 significant ceRNA triplets (*P* < 0.01). (B) Histogram of number of ceRNA partners for a ceRNA in the network. (C) Histogram of number of bridged ceRNA triplets by a miRNA. (PDF 2423 kb)
Additional file 3: Table S1. List of 1,762 core ceRNA pairs. (PDF 535 kb)
Additional file 4: Table S2. Top overrepresented ceRNAs in the core ceRNA network. (PDF 67 kb)
Additional file 5: Figure S3. Functional subnetworks of the core ceRNA interaction network. (A) Subnetwork of the cluster of plasma membrane, extracted from Fig. 2b of main text. (B) Subnetwork of the cluster of intracellular transport, extracted from Fig. 2b of main text. (PDF 489 kb)
Additional file 6: Table S3. Top functional clusters of core ceRNAs. (PDF 2280 kb)
Additional file 7: Figure S4. Validation of core ce-triplets using CGGA dataset. We compared the cumulative distribution curves of interaction scores in the CGGA dataset between 1,762 core ce-triplets identified in TCGA and all other putative ce-triplets. Statistical significance was assessed by the Kolmogorov–Smirnov test. (PDF 162 kb)
Additional file 8: Table S4. Prognostic ceRNA triplets. (PDF 925 kb)
Additional file 9: Figure S5. Prognostic significance of *CCL22* − *IL2RB* − *IRF4* in validation datasets. (A) Distributions of average *t*-scores of the three ceRNA pairs in the optimized group of samples and others in 1,000 iterations. (B) Distributions of median survival in the optimized group of samples and others in 1,000 iterations. (PDF 1295 kb)

## Abbreviations

CEIDCA: ceRNA inference by differential correlation analysis
ce-pair: ceRNA pair
ceRNA: Competing endogenous RNA
ce-triplet: ceRNA triplet
CMI: Conditional mutual information
DAVID: Database for Annotation, Visualization and Integrated Discovery
GBM: Glioblastoma multiforme
IPA: Ingenuity Pathway Analysis
MI: Mutual information
miRNA: microRNA
OS: Overall survival
SMI: Substituted mutual information
TCGA: The Cancer Genome Atlas
