# Supplementary figures and images for: Differential correlation analysis of glioblastoma reveals immune ceRNA interactions predictive of patient survival
Source: BMC Bioinformatics. 2017 Feb 28;18:132. doi: 10.1186/s12859-017-1557-4 (PMC5330036; doi:10.1186/s12859-017-1557-4)

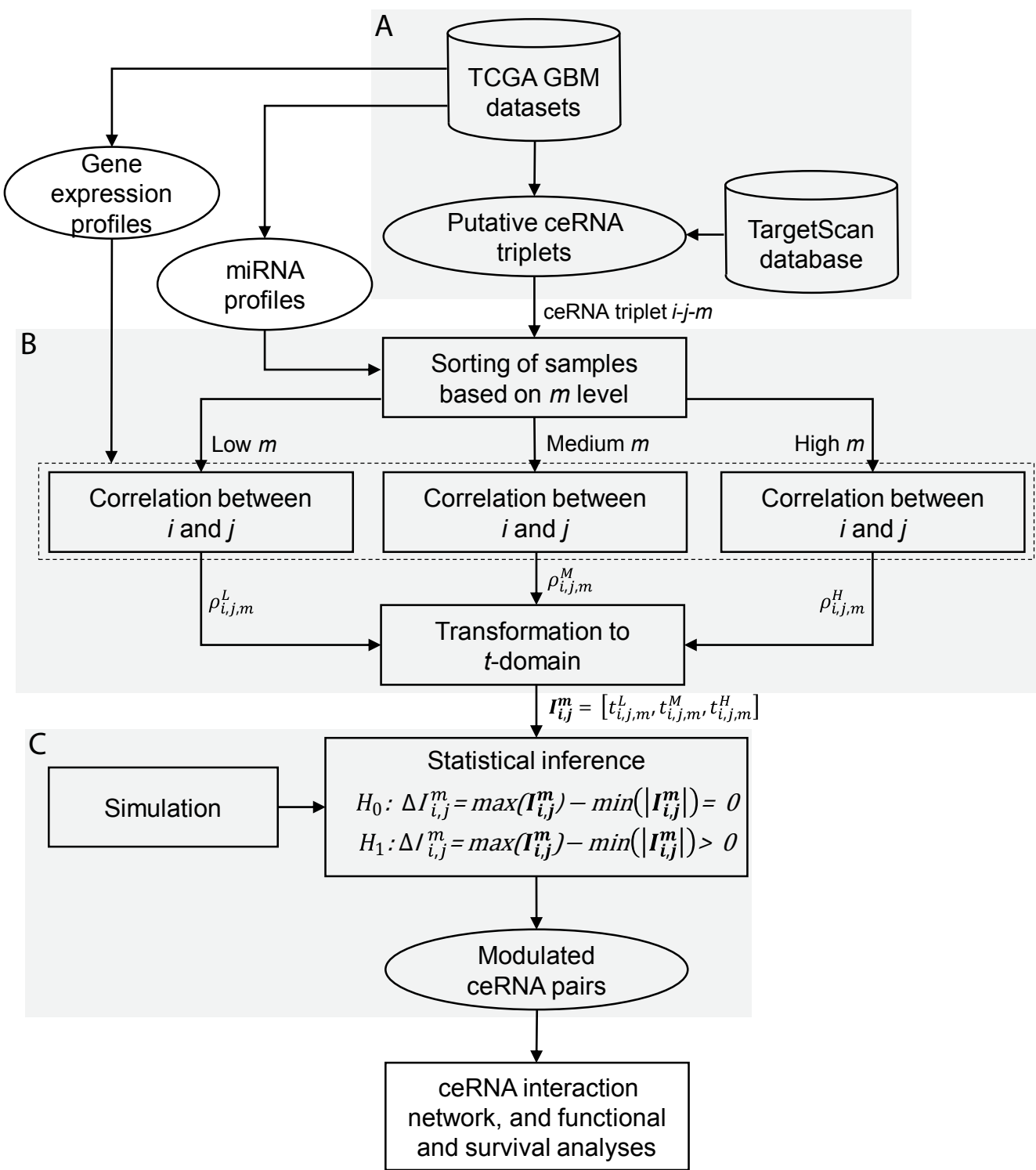

Supplement: Additional file 1: Figure S1. — Flowchart of CEIDCA. The algorithm is mainly built on three components. (A) Definition of putative ceRNA triplets. We defined putative ceRNA triplets by reprocessing prediction miRNA-target data of TargetScan. (B) Measuring interaction strengths of ceRNA pairs. For each putative ceRNA triplet, GBM samples were sorted and divided into three equally-sized groups based on the expression of miRNA. We employed Pearson correlation coefficients and conversion to the t-domain to measure the interaction strength between two ceRNAs. (C) Statistical inference of miRNA-modulated ceRNA pairs. We tested whether a putative ceRNA pair exhibited intensified correlation in one group compared to another. The statistical significance was assessed against a trillion-time simulation. (PDF 367 kb) [file 12859_2017_1557_MOESM1_ESM.pdf]

A

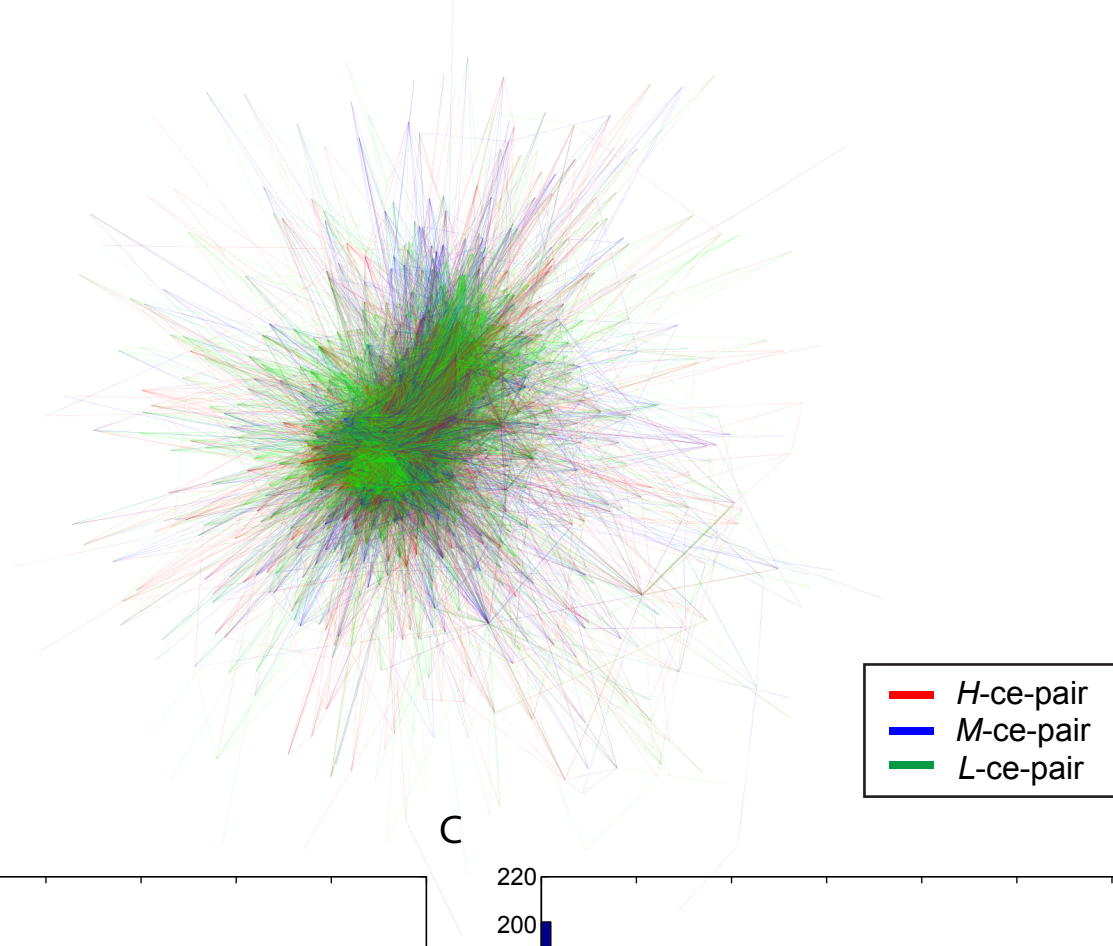

B

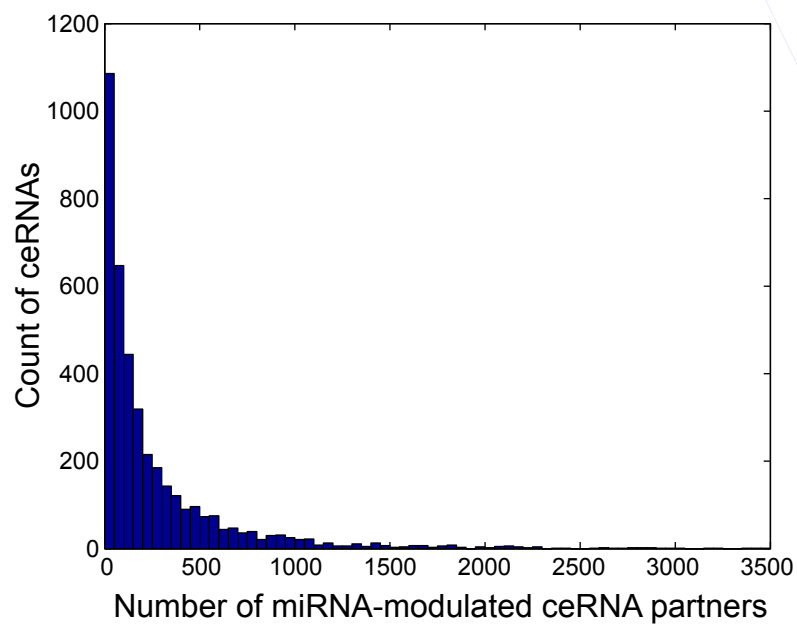

C

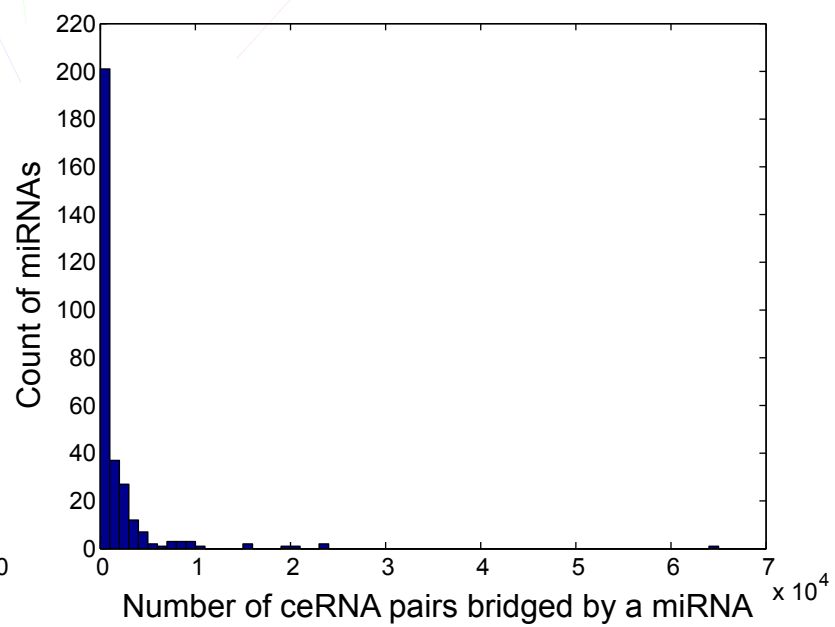

Supplement: Additional file 2: Figure S2. — Complete ceRNA interaction network. (A) ceRNA interaction network constructed by merging 537,304 significant ceRNA triplets (P < 0.01). (B) Histogram of number of ceRNA partners for a ceRNA in the network. (C) Histogram of number of bridged ceRNA triplets by a miRNA. (PDF 2423 kb) [file 12859_2017_1557_MOESM2_ESM.pdf]

A

## I. Cluster of plasma membrane

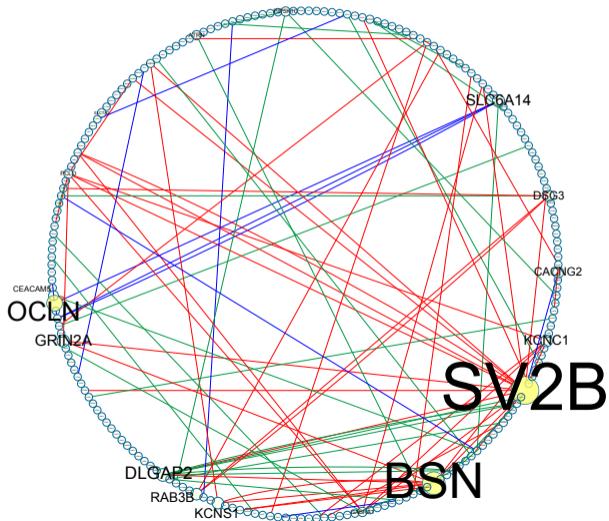

B

## II. Cluster of intracellular transport

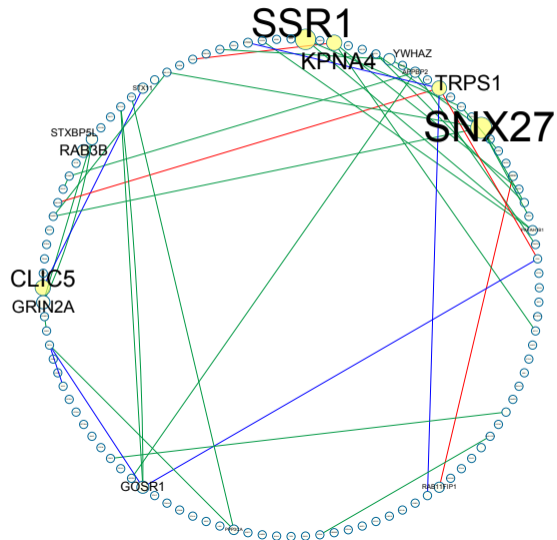

Supplement: Additional file 5: Figure S3. — Functional subnetworks of the core ceRNA interaction network. (A) Subnetwork of the cluster of plasma membrane, extracted from Fig. 2b of main text. (B) Subnetwork of the cluster of intracellular transport, extracted from Fig. 2b of main text. (PDF 489 kb) [file 12859_2017_1557_MOESM5_ESM.pdf]

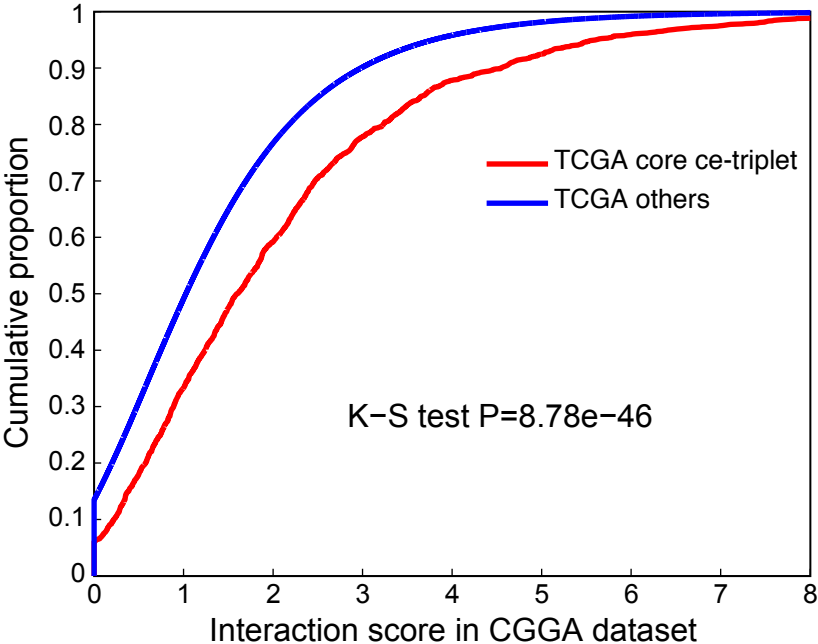

Supplement: Additional file 7: Figure S4. — Validation of core ce-triplets using CGGA dataset. We compared the cumulative distribution curves of interaction scores in the CGGA dataset between 1,762 core ce-triplets identified in TCGA and all other putative ce-triplets. Statistical significance was assessed by the Kolmogorov–Smirnov test. (PDF 162 kb) [file 12859_2017_1557_MOESM7_ESM.pdf]

A

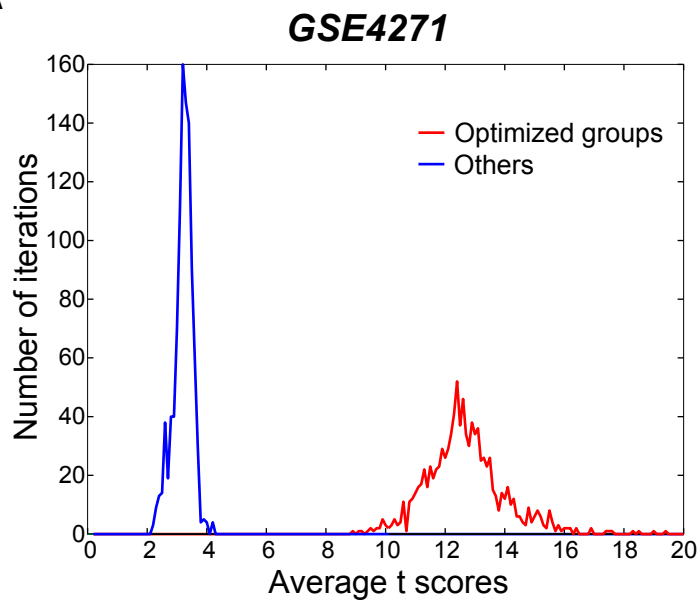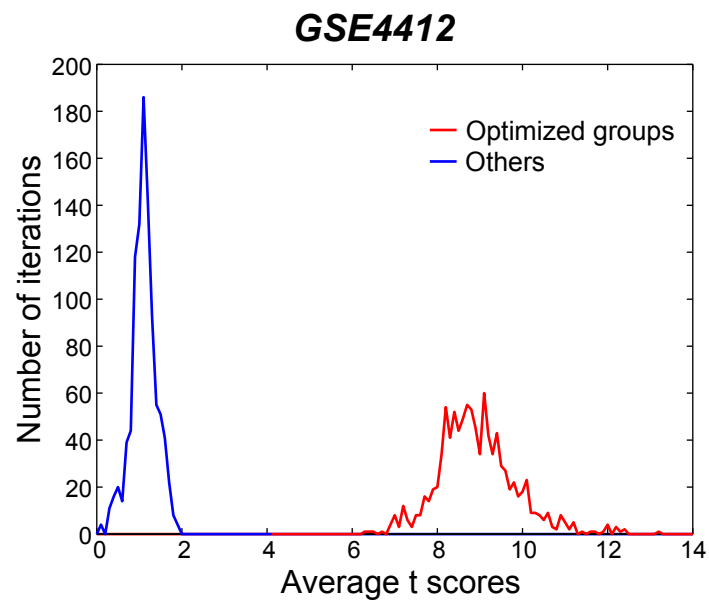

B

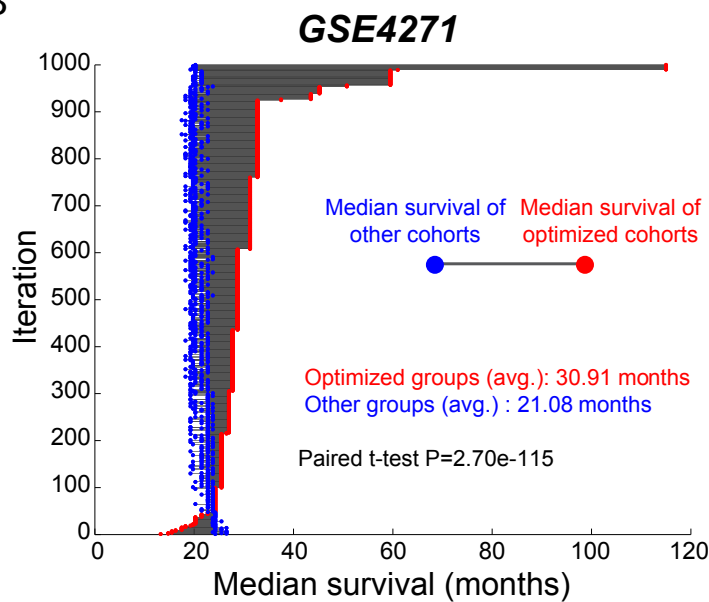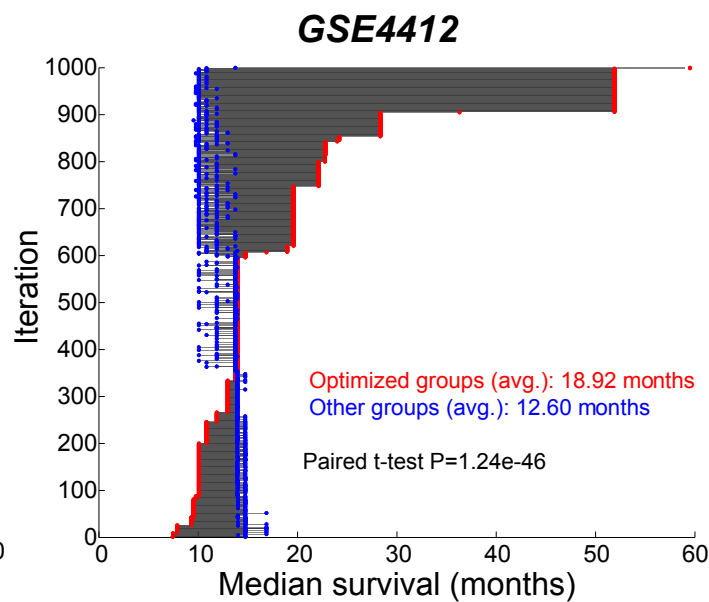

Supplement: Additional file 9: Figure S5. — Prognostic significance of CCL22 − IL2RB − IRF4 in validation datasets. (A) Distributions of average t-scores of the three ceRNA pairs in the optimized group of samples and others in 1,000 iterations. (B) Distributions of median survival in the optimized group of samples and others in 1,000 iterations. (PDF 1295 kb) [file 12859_2017_1557_MOESM9_ESM.pdf]
