# Supplementary material for: Differential correlation analysis of glioblastoma reveals immune ceRNA interactions predictive of patient survival
Source: BMC Bioinformatics. 2017 Feb 28;18:132. doi: 10.1186/s12859-017-1557-4 (PMC5330036; doi:10.1186/s12859-017-1557-4)
Supplement: Additional file 3: Table S1. — List of 1,762 core ceRNA pairs. (PDF 535 kb) [file 12859_2017_1557_MOESM3_ESM.pdf]

Table S1. List of 1,762 core ceRNA pairs

ceRNA1	ceRNA2	miRNA	Optimized group
IRF4	*LTA*	hsa-miR-203	Low miRNA
CCDC68	*SLC6A14*	hsa-miR-340	Medium miRNA
DAZ1	*DAZ4*	hsa-miR-527	Low miRNA
CPB2	*CLIC5*	hsa-miR-143	Medium miRNA
MUC13	*ADIPOQ*	hsa-miR-485-5p	Low miRNA
C1orf116	*EHF*	hsa-miR-539	Low miRNA
TMPRSS4	*KLK10*	hsa-miR-612	High miRNA
CLIC5	*CCRL1*	hsa-miR-340	Medium miRNA
STX11	*CLDN18*	hsa-miR-34a	High miRNA
C1orf116	*GPA33*	hsa-miR-147	Medium miRNA
CCDC68	*CLIC5*	hsa-miR-340	High miRNA
DSC3	*CEACAM5*	hsa-miR-561	Low miRNA
VGLL3	*SLC6A14*	hsa-miR-587	High miRNA
SV2B	*MYT1L*	hsa-miR-495	High miRNA
C1orf116	*VGLL3*	hsa-miR-369-3p	Medium miRNA
VGLL3	*CLIC5*	hsa-miR-579	Low miRNA
CLIC5	*OCLN*	hsa-miR-340	Medium miRNA
DAZ1	*DAZ4*	hsa-miR-200b	Low miRNA
GPM6A	*PMP2*	hsa-miR-369-3p	High miRNA
DAZ1	*DAZ4*	hsa-miR-577	High miRNA
KIAA1045	*KCNS1*	hsa-miR-326	High miRNA
OCLN	*SLC6A14*	hsa-miR-340	Medium miRNA
CLEC2D	*IRF4*	hsa-miR-587	Medium miRNA
SV2B	*GABRA1*	hsa-miR-203	High miRNA
VGLL3	*CLIC5*	hsa-miR-340	Medium miRNA
IRF4	*CCL22*	hsa-miR-34a	Low miRNA
DAZ1	*DAZ4*	hsa-miR-200c	High miRNA
FAT2	*PLD1*	hsa-miR-519e	High miRNA
CCDC68	*LMO7*	hsa-miR-340	Medium miRNA
BSN	*FBXO41*	hsa-miR-149	High miRNA
RAB3B	*PKP1*	hsa-miR-558	High miRNA
IRF4	*CCL22*	hsa-miR-449b	Medium miRNA
HPCAL4	*SV2B*	hsa-miR-7	High miRNA

LAMP3	*RAB11FIP1*	hsa-miR-519d	Medium miRNA
MDM4	*PLEKHA6*	hsa-miR-622	Medium miRNA
KIAA1045	*CNNM1*	hsa-miR-326	High miRNA
POU2AF1	*GRHL2*	hsa-miR-766	Medium miRNA
G6PC	*SLC38A4*	hsa-miR-607	High miRNA
KIAA0513	*AAK1*	hsa-miR-485-5p	High miRNA
ADARB2	*SLCO1A2*	hsa-miR-579	Low miRNA
VGLL3	*AGTR2*	hsa-miR-607	Medium miRNA
SPARC	*TCF4*	hsa-miR-203	High miRNA
SSBP3	*TRIM3*	hsa-miR-608	Low miRNA
CEACAM5	*NPR3*	hsa-miR-607	High miRNA
VGLL3	*CLIC5*	hsa-miR-186	Low miRNA
GOSR1	*ANKRD12*	hsa-miR-150	Medium miRNA
SSBP3	*ATP6V0A1*	hsa-miR-608	Low miRNA
OCLN	*TMEM30B*	hsa-miR-561	Medium miRNA
OCLN	*CEACAM5*	hsa-miR-607	High miRNA
MBNL3	*SLC2A2*	hsa-miR-570	Low miRNA
CLIC5	*SLC6A14*	hsa-miR-340	Medium miRNA
VGLL3	*TMPRSS2*	hsa-miR-579	Low miRNA
SV2B	*AAK1*	hsa-miR-136	High miRNA
FAT2	*PLD1*	hsa-miR-515-3p	Medium miRNA
KCNS1	*CHD5*	hsa-miR-149	High miRNA
RNF4	*PPP3CA*	hsa-miR-495	Low miRNA
PRRG4	*DSG3*	hsa-miR-607	High miRNA
STEAP4	*NPR3*	hsa-miR-340	Low miRNA
PRRG4	*RAB11FIP1*	hsa-miR-20a	Medium miRNA
NEFL	*GABRA1*	hsa-miR-203	High miRNA
CACNG2	*SLC8A2*	hsa-miR-637	Low miRNA
MDM4	*ETNK2*	hsa-miR-150	High miRNA
SLC12A5	*DLGAP2*	hsa-miR-25	Low miRNA
VGLL3	*ADAMTSL3*	hsa-miR-340	Medium miRNA
AAK1	*SCN2A*	hsa-miR-107	High miRNA
FGD6	*DSG3*	hsa-miR-369-3p	High miRNA
PCSK2	*AAK1*	hsa-miR-203	High miRNA
PTPRB	*VGLL3*	hsa-miR-23a	Low miRNA
SLC8A2	*SV2B*	hsa-miR-7	High miRNA

STEAP4	*SLC6A14*	hsa-miR-340	Medium miRNA
KCNS1	*CAMK2A*	hsa-miR-326	High miRNA
LMO7	*SLC6A14*	hsa-miR-105	Medium miRNA
OCLN	*CCRL1*	hsa-miR-340	Medium miRNA
SYT11	*GPM6A*	hsa-miR-186	Medium miRNA
SLC8A2	*PAK6*	hsa-miR-185	High miRNA
PAX1	*COL11A2*	hsa-miR-608	Medium miRNA
IRF4	*IL2RB*	hsa-miR-449b	Medium miRNA
KCNC1	*AAK1*	hsa-miR-203	High miRNA
KIAA1045	*CHD5*	hsa-miR-370	High miRNA
SERBP1	*PPP3CA*	hsa-miR-495	Low miRNA
HELZ	*SNX27*	hsa-miR-607	Low miRNA
KIAA1045	*IQSEC3*	hsa-miR-637	Low miRNA
FAM46C	*FUT1*	hsa-miR-515-5p	High miRNA
STX11	*VGLL3*	hsa-miR-568	High miRNA
VGLL3	*OCLN*	hsa-miR-340	Medium miRNA
KLK10	*PKP1*	hsa-miR-661	Low miRNA
AAK1	*CAMKV*	hsa-miR-329	High miRNA
CTSL2	*SERPINB13*	hsa-miR-570	High miRNA
C1orf116	*STX11*	hsa-miR-539	High miRNA
CCDC68	*VGLL3*	hsa-miR-340	Medium miRNA
PAX8	*KCNK15*	hsa-miR-637	Medium miRNA
STX11	*VGLL3*	hsa-miR-494	Low miRNA
PTPRB	*AGTR2*	hsa-miR-607	High miRNA
PACS2	*CDC42BPB*	hsa-miR-608	Low miRNA
SH3TC2	*SEC14L5*	hsa-miR-138	High miRNA
SLC8A2	*FAIM2*	hsa-miR-7	High miRNA
VGLL3	*CLIC5*	hsa-miR-511	Medium miRNA
AAK1	*CHD5*	hsa-miR-504	High miRNA
CCDC68	*AGTR2*	hsa-miR-607	Medium miRNA
NCDN	*SYP*	hsa-miR-608	Low miRNA
NPR3	*ATP8A1*	hsa-miR-607	High miRNA
HOOK1	*NPR3*	hsa-miR-607	High miRNA
CREB1	*LRPPRC*	hsa-miR-607	Low miRNA
KIAA1045	*KIAA0319*	hsa-miR-326	High miRNA
SLC12A3	*SLC7A2*	hsa-miR-661	Low miRNA

PAX8	*PAX2*	hsa-miR-637	High miRNA
SLC8A2	*CNNM1*	hsa-miR-608	Low miRNA
CLIC5	*OCLN*	hsa-miR-186	Low miRNA
FBXO41	*CHD5*	hsa-miR-149	High miRNA
CHML	*CPT1A*	hsa-miR-579	High miRNA
TNFRSF1B	*EMR2*	hsa-miR-150	Low miRNA
GPA33	*VGLL3*	hsa-miR-147	Medium miRNA
CEACAM5	*EHF*	hsa-miR-607	Low miRNA
FGD6	*DSG3*	hsa-miR-607	High miRNA
STEAP4	*VGLL3*	hsa-miR-656	Medium miRNA
CNTN2	*SH3TC2*	hsa-miR-185	High miRNA
C1orf116	*VGLL3*	hsa-miR-147	Medium miRNA
SLC12A5	*SNCB*	hsa-miR-663	Low miRNA
FGF7	*DCBLD2*	hsa-miR-340	High miRNA
KIF5C	*RAP2A*	hsa-miR-186	Medium miRNA
CLCA2	*PRRG4*	hsa-miR-495	High miRNA
CNTN2	*CLMN*	hsa-miR-185	High miRNA
KCNS1	*KIAA0319*	hsa-miR-326	High miRNA
KIAA1045	*SLC8A2*	hsa-miR-637	Medium miRNA
TNFSF15	*VGLL3*	hsa-miR-570	Medium miRNA
KCNC1	*PCLO*	hsa-miR-203	High miRNA
KIAA0513	*AAK1*	hsa-miR-107	High miRNA
GALNT6	*ST3GAL1*	hsa-miR-24	Medium miRNA
MAPKAPK2	*ARHGDIA*	hsa-miR-637	Low miRNA
KCNC1	*PCSK2*	hsa-miR-203	High miRNA
STEAP4	*CCDC68*	hsa-miR-340	Medium miRNA
OCLN	*CEACAM5*	hsa-miR-561	Medium miRNA
ADAMTSL3	*SLC6A14*	hsa-miR-340	Medium miRNA
SV2B	*FAIM2*	hsa-miR-7	High miRNA
ARRB1	*VGLL3*	hsa-miR-587	High miRNA
DSC3	*PRRG4*	hsa-miR-561	High miRNA
NOS1AP	*PCSK2*	hsa-miR-329	High miRNA
SV2B	*NRIP3*	hsa-miR-539	High miRNA
SV2B	*NEFL*	hsa-miR-203	High miRNA
TRHDE	*AAK1*	hsa-miR-580	Low miRNA
TRPS1	*CEP68*	hsa-miR-204	Medium miRNA

AAK1	*CAMK2A*	hsa-miR-485-5p	High miRNA
SLC8A2	*AAK1*	hsa-miR-7	High miRNA
RASAL1	*SV2B*	hsa-miR-769-5p	High miRNA
LMO7	*ADAMTSL3*	hsa-miR-340	Medium miRNA
OSBPL2	*SERINC1*	hsa-miR-607	Low miRNA
PAQR5	*VGLL3*	hsa-miR-607	High miRNA
RAB6B	*AAK1*	hsa-miR-485-5p	High miRNA
RAB11FIP1	*KLRD1*	hsa-miR-519d	Medium miRNA
CEACAM5	*SLC38A4*	hsa-miR-607	Low miRNA
CBX5	*MAP1B*	hsa-miR-616	High miRNA
SSR1	*RAP2C*	hsa-miR-607	Low miRNA
POU2AF1	*LTA*	hsa-miR-203	Low miRNA
FBXO41	*KIAA0319*	hsa-miR-326	High miRNA
KIAA1045	*FBXO41*	hsa-miR-326	High miRNA
FBXO41	*AAK1*	hsa-miR-149	High miRNA
KCNS1	*AAK1*	hsa-miR-433	High miRNA
SLC38A4	*EHF*	hsa-miR-607	Low miRNA
CACNG2	*CNNM1*	hsa-miR-608	Low miRNA
AAK1	*AKAP11*	hsa-miR-329	High miRNA
DAZ1	*DAZ4*	hsa-miR-561	High miRNA
TNFRSF13B	*MS4A1*	hsa-miR-593	High miRNA
CYB5B	*RAB14*	hsa-miR-577	Low miRNA
ICK	*ZEB2*	hsa-miR-607	Low miRNA
RAB3B	*DSG3*	hsa-miR-580	High miRNA
KIF1B	*PMP2*	hsa-miR-599	High miRNA
SNX27	*ATRN*	hsa-miR-607	Low miRNA
SCN9A	*DSG3*	hsa-miR-607	High miRNA
PCLO	*CNTNAP2*	hsa-miR-218	High miRNA
TBL1XR1	*PIP4K2B*	hsa-miR-105	Low miRNA
LMO7	*SLC6A14*	hsa-miR-340	Medium miRNA
ZBTB38	*ARL4C*	hsa-miR-101	Medium miRNA
GOSR2	*LARP4*	hsa-miR-603	Low miRNA
GABRA1	*PCLO*	hsa-miR-203	High miRNA
CREB1	*TOMM70A*	hsa-miR-607	Low miRNA
SV2B	*AAK1*	hsa-miR-7	High miRNA
SERPINB13	*SLC6A14*	hsa-miR-340	Low miRNA

FGF7	*FAM46C*	hsa-miR-607	Low miRNA
STX11	*CLDN18*	hsa-miR-495	Medium miRNA
NCDN	*CNNM1*	hsa-miR-608	Low miRNA
SH3TC2	*CLMN*	hsa-miR-185	High miRNA
NCDN	*IQSEC3*	hsa-miR-637	Low miRNA
SNPH	*AAK1*	hsa-miR-485-5p	High miRNA
SGPP1	*KPNA4*	hsa-miR-561	Low miRNA
ITGAM	*PTPRC*	hsa-miR-603	Low miRNA
MBNL3	*SLC38A4*	hsa-miR-607	High miRNA
CCDC68	*FMO2*	hsa-miR-340	Medium miRNA
CACNG2	*AAK1*	hsa-miR-578	Low miRNA
STEAP4	*VGLL3*	hsa-miR-369-3p	Low miRNA
OCLN	*FBN1*	hsa-miR-488	High miRNA
CLDN1	*CLIC5*	hsa-miR-186	Low miRNA
PAFAH1B1	*SSR1*	hsa-miR-603	Low miRNA
RAB3B	*FGD6*	hsa-miR-186	Medium miRNA
CEACAM5	*TMEM30B*	hsa-miR-607	High miRNA
BSN	*DLGAP2*	hsa-miR-539	High miRNA
CACNG2	*HECW1*	hsa-miR-578	Low miRNA
ARRB1	*SLC34A2*	hsa-miR-637	Low miRNA
WDFY3	*ATRN*	hsa-miR-544	Low miRNA
NRIP3	*CAMK2A*	hsa-miR-485-5p	High miRNA
PAX8	*CLIC5*	hsa-miR-511	Low miRNA
NR4A3	*STEAP4*	hsa-miR-641	High miRNA
BSN	*PAK6*	hsa-miR-185	High miRNA
KIAA1045	*GRIN2A*	hsa-miR-326	High miRNA
CANX	*CUL3*	hsa-miR-186	Medium miRNA
HELZ	*NIPBL*	hsa-miR-544	Low miRNA
ADAMTSL3	*KCNJ13*	hsa-miR-340	Low miRNA
RUNX3	*RAC2*	hsa-miR-608	Medium miRNA
STX11	*VGLL3*	hsa-miR-559	Medium miRNA
SYNGR3	*SLC17A7*	hsa-miR-185	High miRNA
KPNA4	*MAP3K2*	hsa-miR-607	Low miRNA
RASGRF1	*STX11*	hsa-miR-661	Medium miRNA
ZMIZ1	*TSC1*	hsa-miR-637	Low miRNA
OSBPL2	*AKAP11*	hsa-miR-607	Low miRNA

KIAA1045	*CACNB4*	hsa-miR-218	High miRNA
MEF2C	*SV2B*	hsa-miR-203	High miRNA
GIPC2	*EHF*	hsa-miR-607	Low miRNA
CLEC2D	*SLC38A4*	hsa-miR-607	High miRNA
ATXN1	*TCF4*	hsa-miR-203	High miRNA
ALDH1A2	*GFRA1*	hsa-miR-542-3p	High miRNA
MDM4	*ETNK2*	hsa-miR-608	High miRNA
NCDN	*SLC8A2*	hsa-miR-637	Low miRNA
SV2B	*CACNB4*	hsa-miR-7	High miRNA
UBTF	*SSBP3*	hsa-miR-608	Low miRNA
VPS24	*ACTR2*	hsa-miR-495	Low miRNA
UBE4B	*ASB1*	hsa-miR-607	Low miRNA
CLCN4	*UBE4B*	hsa-miR-607	Low miRNA
RAB5B	*PIP5K1C*	hsa-miR-661	Low miRNA
PRPF40A	*UBE2G1*	hsa-miR-539	Low miRNA
EREG	*CPA4*	hsa-miR-769-5p	Medium miRNA
DDX3Y	*NLGN4Y*	hsa-miR-561	Low miRNA
APPL1	*ICK*	hsa-miR-607	Low miRNA
NR4A3	*CLIC5*	hsa-miR-641	High miRNA
ATP2B4	*ZC3H11A*	hsa-miR-186	Medium miRNA
PAFAH1B1	*JHDM1D*	hsa-miR-600	Low miRNA
KCNJ12	*TRPM8*	hsa-miR-603	Low miRNA
SV2B	*NRIP3*	hsa-miR-485-5p	High miRNA
MEF2C	*GABRA1*	hsa-miR-203	High miRNA
BSN	*CHD5*	hsa-miR-149	High miRNA
PAK6	*SLC17A7*	hsa-miR-185	High miRNA
PRPF4B	*ASB1*	hsa-miR-607	Low miRNA
SSR1	*C12orf5*	hsa-miR-603	Low miRNA
MEOX1	*PAX2*	hsa-miR-765	Low miRNA
SLC8A2	*IQSEC3*	hsa-miR-637	Low miRNA
BSN	*SYP*	hsa-miR-608	Low miRNA
KCNC1	*AAK1*	hsa-miR-605	Low miRNA
UBE4A	*TCF4*	hsa-miR-142-5p	High miRNA
STYK1	*DSG3*	hsa-miR-204	Low miRNA
GIMAP6	*RAC2*	hsa-miR-608	Low miRNA
IRF4	*CLDN18*	hsa-miR-34a	Medium miRNA

PPM1F	*PIP5K1C*	hsa-miR-608	Low miRNA
PRRG4	*FGD6*	hsa-miR-607	Medium miRNA
KCNC1	*FBXO41*	hsa-miR-149	High miRNA
BSN	*KCNS1*	hsa-miR-149	High miRNA
CCDC68	*OCLN*	hsa-miR-340	Medium miRNA
TRIM33	*TCF4*	hsa-miR-629	Low miRNA
APC	*CTNND2*	hsa-miR-561	High miRNA
MCTP2	*LGALS8*	hsa-miR-607	Medium miRNA
SV2B	*AAK1*	hsa-miR-495	High miRNA
COL4A4	*CCDC68*	hsa-miR-340	Medium miRNA
AGPS	*KPNA4*	hsa-miR-607	Low miRNA
TCF4	*SOBP*	hsa-miR-579	High miRNA
DSC3	*SLC6A14*	hsa-miR-105	Low miRNA
CLDN18	*CLIC5*	hsa-miR-659	Low miRNA
HELZ	*ARHGAP5*	hsa-miR-607	Low miRNA
PTPRB	*CLIC5*	hsa-miR-579	High miRNA
SV2B	*RAB6B*	hsa-miR-485-5p	High miRNA
ARRB1	*CCL21*	hsa-miR-608	Low miRNA
PAFAH1B1	*GSPT1*	hsa-miR-603	Low miRNA
CLIC5	*LMO7*	hsa-miR-340	Medium miRNA
LPHN1	*TRPS1*	hsa-miR-204	High miRNA
PKP1	*EPN3*	hsa-miR-661	Medium miRNA
TBL1XR1	*KPNA6*	hsa-miR-570	Low miRNA
ICK	*ASB1*	hsa-miR-607	Low miRNA
NEFL	*PCSK2*	hsa-miR-203	High miRNA
RAB11A	*SSR1*	hsa-miR-520h	Low miRNA
SYNGR3	*BSN*	hsa-miR-185	High miRNA
SEC16A	*GOSR1*	hsa-miR-106a	Low miRNA
SEC16A	*GOSR1*	hsa-miR-20b	Low miRNA
TARDBP	*SNX27*	hsa-miR-607	Low miRNA
RAP2B	*TM9SF3*	hsa-miR-142-5p	High miRNA
BSN	*FBXO41*	hsa-miR-185	High miRNA
APPL1	*ARHGAP5*	hsa-miR-607	Low miRNA
HPCAL4	*AAK1*	hsa-miR-7	High miRNA
KCNC1	*NEFL*	hsa-miR-203	High miRNA
SNX19	*ARHGAP5*	hsa-miR-607	Low miRNA

CYLD	*NANOS1*	hsa-miR-544	Low miRNA
TBL1XR1	*KPNA4*	hsa-miR-570	Low miRNA
ARHGAP5	*PTPN11*	hsa-miR-607	Low miRNA
STXBP5L	*GRIN2A*	hsa-miR-105	Low miRNA
NCDN	*SLC8A2*	hsa-miR-608	Low miRNA
SCN3B	*KIAA0513*	hsa-miR-485-5p	High miRNA
TRAF1	*IL2RB*	hsa-miR-214	Low miRNA
LAMP3	*RAB11FIP1*	hsa-miR-93	High miRNA
BSN	*FBXO41*	hsa-miR-326	High miRNA
G6PC	*SH2D1A*	hsa-miR-607	High miRNA
SNX19	*GPR107*	hsa-miR-587	Low miRNA
POU4F2	*ONECUT2*	hsa-miR-185	High miRNA
SH3TC2	*ADARB2*	hsa-miR-608	Low miRNA
KIAA1045	*DLGAP2*	hsa-miR-637	Low miRNA
STX11	*CLDN18*	hsa-miR-449b	Low miRNA
RAB3B	*MCTP2*	hsa-miR-203	High miRNA
SLC8A2	*AAK1*	hsa-miR-637	Low miRNA
RAB14	*APPL1*	hsa-miR-607	Low miRNA
SNX27	*PRPF4B*	hsa-miR-607	Low miRNA
VGLL3	*CCRL1*	hsa-miR-340	Medium miRNA
CCDC68	*SLC22A3*	hsa-miR-142-5p	Medium miRNA
OCLN	*ADAMTSL3*	hsa-miR-340	Medium miRNA
HECA	*OSBPL2*	hsa-miR-607	Low miRNA
PRSS16	*C1orf116*	hsa-miR-539	Low miRNA
OGN	*OCLN*	hsa-miR-488	High miRNA
INCENP	*SLC7A2*	hsa-miR-15b	Medium miRNA
SLC22A3	*AGTR2*	hsa-miR-607	High miRNA
SV2B	*PCLO*	hsa-miR-203	High miRNA
TRAF3	*MS4A1*	hsa-miR-613	Low miRNA
PTPRT	*GRIN2A*	hsa-miR-203	High miRNA
EIF5A2	*FAM46C*	hsa-miR-106a	Medium miRNA
RAB3B	*ST8SIA3*	hsa-miR-186	Low miRNA
RTF1	*EP400*	hsa-miR-603	Low miRNA
SLC8A2	*BSN*	hsa-miR-185	High miRNA
ZEB2	*RPS6KB1*	hsa-miR-200b	High miRNA
SNX27	*YTHDC1*	hsa-miR-607	Low miRNA

IQSEC3	*SNCB*	hsa-miR-663	Low miRNA
TNPO1	*PPP3CA*	hsa-miR-495	Low miRNA
KIAA0513	*CAMK2A*	hsa-miR-485-5p	High miRNA
ACSL4	*GFPT1*	hsa-miR-181b	Medium miRNA
MTF1	*ARHGAP5*	hsa-miR-607	Low miRNA
HPCAL4	*SLC8A2*	hsa-miR-7	High miRNA
KCNS1	*CNNM1*	hsa-miR-326	High miRNA
ATXN1	*AUTS2*	hsa-miR-527	Medium miRNA
OCLN	*NPR3*	hsa-miR-340	Low miRNA
STXBP5L	*AAK1*	hsa-miR-605	Low miRNA
MECP2	*TMEM57*	hsa-miR-141	High miRNA
PCSK2	*AAK1*	hsa-miR-329	High miRNA
DCP1A	*YWHAZ*	hsa-miR-544	Low miRNA
CUL4B	*SSR3*	hsa-miR-570	Low miRNA
C1orf116	*DSG3*	hsa-miR-369-3p	High miRNA
RAB5A	*RAB11A*	hsa-miR-539	Low miRNA
SENP5	*DSG3*	hsa-miR-607	High miRNA
WDFY3	*YWHAZ*	hsa-miR-544	Low miRNA
SLC6A14	*TP63*	hsa-miR-340	Low miRNA
MBNL3	*SH2D1A*	hsa-miR-607	High miRNA
KIAA1045	*PTPRT*	hsa-miR-636	Low miRNA
SNX27	*MTDH*	hsa-miR-607	Low miRNA
PCM1	*PDLIM5*	hsa-miR-150	Medium miRNA
PIP4K2B	*PIP5K1C*	hsa-miR-661	Low miRNA
AAK1	*NDRG3*	hsa-miR-485-5p	High miRNA
CLDN8	*PLD1*	hsa-miR-579	Medium miRNA
PCSK2	*GABRA1*	hsa-miR-203	High miRNA
SV2B	*KIAA0513*	hsa-miR-485-5p	High miRNA
ATP2B2	*AAK1*	hsa-miR-7	High miRNA
CACNG2	*AAK1*	hsa-miR-637	Low miRNA
SLC7A2	*TFAP2C*	hsa-miR-603	High miRNA
ARL5A	*PMPCB*	hsa-miR-200b	High miRNA
DCP1A	*ZNF629*	hsa-miR-544	High miRNA
AAK1	*PCLO*	hsa-miR-203	High miRNA
LDLR	*VGLL3*	hsa-miR-520g	Low miRNA
GFPT1	*NRIP1*	hsa-miR-656	Low miRNA

SLC17A6	*DLGAP2*	hsa-miR-25	Low miRNA
CLDN1	*KLF5*	hsa-miR-186	Medium miRNA
UCHL5	*UMPS*	hsa-miR-485-3p	Low miRNA
BCL11B	*ONECUT2*	hsa-miR-195	Medium miRNA
GABRA1	*AAK1*	hsa-miR-203	High miRNA
PPP1R16B	*FA2H*	hsa-miR-608	Low miRNA
QKI	*JHDM1D*	hsa-miR-600	Low miRNA
PPP1R16B	*CNNM1*	hsa-miR-31	High miRNA
CREB1	*RBBP4*	hsa-miR-539	Low miRNA
TMEM127	*HGSNAT*	hsa-miR-204	Medium miRNA
ETF1	*ARF6*	hsa-miR-520h	Low miRNA
SYT13	*DLGAP2*	hsa-miR-205	Low miRNA
TNFSF15	*PIGR*	hsa-miR-580	Medium miRNA
SLC7A11	*MTM1*	hsa-miR-570	Low miRNA
SV2B	*CAMK2A*	hsa-miR-485-5p	High miRNA
CCDC68	*CCRL1*	hsa-miR-340	Medium miRNA
SORBS3	*SSBP3*	hsa-miR-608	Low miRNA
RAB11FIP2	*NANOS1*	hsa-miR-200b	Low miRNA
KIAA1045	*ATP2B3*	hsa-miR-326	High miRNA
NOTCH2	*TTC28*	hsa-miR-204	Medium miRNA
QKI	*OSBPL2*	hsa-miR-607	Low miRNA
KCNC1	*SV2B*	hsa-miR-203	High miRNA
GOSR1	*KIAA0226*	hsa-miR-150	Medium miRNA
SV2B	*BSN*	hsa-miR-539	High miRNA
CAMK2G	*AAK1*	hsa-miR-495	High miRNA
FAT2	*BCL11B*	hsa-miR-519e	High miRNA
TFEC	*MGAT4A*	hsa-miR-142-5p	High miRNA
PTER	*ENTPD1*	hsa-miR-607	Medium miRNA
AP1S2	*NLGN4X*	hsa-miR-561	High miRNA
ASB1	*RNF8*	hsa-miR-607	Low miRNA
GRIN2A	*AAK1*	hsa-miR-203	High miRNA
BSN	*KIAA0319*	hsa-miR-326	High miRNA
KIF3A	*SPAG9*	hsa-miR-181b	Low miRNA
APPL1	*IREB2*	hsa-miR-607	Low miRNA
TRPS1	*ELAVL1*	hsa-miR-577	Low miRNA
MAP4K4	*LPGAT1*	hsa-miR-559	Low miRNA

POU2AF1	*CCL22*	hsa-miR-485-5p	High miRNA
PPM1H	*TPPP*	hsa-miR-661	Low miRNA
NRIP2	*ANK1*	hsa-miR-650	Medium miRNA
MECP2	*C17orf63*	hsa-miR-625	Low miRNA
C5AR1	*EMR2*	hsa-miR-150	Low miRNA
STXBP5L	*TRHDE*	hsa-miR-580	Low miRNA
TMOD3	*ZFHX3*	hsa-miR-570	Low miRNA
TRIM2	*PKIA*	hsa-miR-145	High miRNA
CLDN18	*VGLL3*	hsa-miR-495	High miRNA
BSN	*AAK1*	hsa-miR-149	High miRNA
LRPPRC	*C1orf9*	hsa-miR-607	Low miRNA
KCNC1	*GABRA1*	hsa-miR-203	High miRNA
RUNX1T1	*BCL11A*	hsa-miR-186	Low miRNA
GSPT1	*KPNA1*	hsa-miR-577	Low miRNA
OPA3	*PGPEP1*	hsa-miR-612	Low miRNA
BSN	*STXBP5L*	hsa-miR-539	High miRNA
KCTD2	*PIP5K1C*	hsa-miR-608	Low miRNA
SH3TC2	*ADARB2*	hsa-miR-581	Low miRNA
SV2B	*AAK1*	hsa-miR-527	Low miRNA
ZNF430	*C14orf135*	hsa-miR-561	Low miRNA
IL24	*MCTP2*	hsa-miR-203	High miRNA
NLGN4X	*PMP2*	hsa-miR-23a	Medium miRNA
PTGFR	*GFRA1*	hsa-miR-520f	Low miRNA
LHFPL2	*ENTPD1*	hsa-miR-568	Low miRNA
IREB2	*ASB1*	hsa-miR-607	Low miRNA
SV2B	*AAK1*	hsa-miR-485-5p	High miRNA
SYP	*TRIM3*	hsa-miR-608	Low miRNA
NEFH	*STXBP5L*	hsa-miR-137	High miRNA
KIAA0513	*CNTNAP2*	hsa-miR-372	Low miRNA
CDC42EP1	*ARHGDIA*	hsa-miR-637	Low miRNA
SMARCA5	*PPP3CA*	hsa-miR-381	Low miRNA
FGF7	*VGLL3*	hsa-miR-340	High miRNA
ICK	*ZEB2*	hsa-miR-369-3p	Medium miRNA
TMED7	*PEX19*	hsa-miR-561	Low miRNA
PSD3	*ANK2*	hsa-miR-302b	Low miRNA
SERPINB13	*NPR3*	hsa-miR-340	Medium miRNA

TBL1XR1	*MTF1*	hsa-miR-367	Low miRNA
RBMS2	*IRAK3*	hsa-miR-641	Low miRNA
MARK2	*SSBP3*	hsa-miR-608	Low miRNA
DAZ1	*DAZ4*	hsa-miR-429	Medium miRNA
RAB3B	*KLK10*	hsa-miR-612	High miRNA
C1orf116	*DPT*	hsa-miR-299-3p	Low miRNA
PAX8	*SLC34A2*	hsa-miR-637	Medium miRNA
SLC7A2	*GFRA1*	hsa-miR-539	Low miRNA
PTPRT	*AAK1*	hsa-miR-203	High miRNA
PPP1R12A	*CPSF6*	hsa-miR-607	Low miRNA
CDC42BPB	*SSBP3*	hsa-miR-608	Low miRNA
AGPS	*ZEB2*	hsa-miR-200a	High miRNA
VGLL3	*OCLN*	hsa-miR-520h	Medium miRNA
GFRA1	*PDE3B*	hsa-miR-186	Medium miRNA
AGPS	*KPNA4*	hsa-miR-656	Low miRNA
MARK2	*ATP6V0A1*	hsa-miR-608	Low miRNA
IRF4	*IL2RB*	hsa-miR-34a	Low miRNA
RAB3B	*SERPINB13*	hsa-miR-570	High miRNA
PRKACA	*SSBP3*	hsa-miR-608	Low miRNA
TGOLN2	*RANBP2*	hsa-miR-607	Low miRNA
NR4A3	*SLC18A2*	hsa-miR-641	High miRNA
ATAD2B	*ASB1*	hsa-miR-607	Low miRNA
SCN9A	*PRRG4*	hsa-miR-607	High miRNA
SLC8A2	*CAMKK2*	hsa-miR-7	High miRNA
SCRN1	*KIAA0494*	hsa-miR-659	Low miRNA
GPI	*GLG1*	hsa-miR-661	High miRNA
QKI	*MIA3*	hsa-miR-186	Medium miRNA
RAB14	*SNX27*	hsa-miR-607	Low miRNA
SGPP1	*EXOC5*	hsa-miR-561	Low miRNA
AAK1	*HLF*	hsa-miR-520g	Low miRNA
NOS1AP	*AAK1*	hsa-miR-329	High miRNA
FECH	*ULK2*	hsa-miR-607	Low miRNA
TARDBP	*ZC3H11A*	hsa-miR-186	High miRNA
SV2B	*PCSK2*	hsa-miR-203	High miRNA
DAZL	*SGCD*	hsa-miR-577	Low miRNA
PPM1H	*FAIM2*	hsa-miR-661	Low miRNA

B3GAT1	*ASTN1*	hsa-miR-147	High miRNA
STX12	*SERINC1*	hsa-miR-607	High miRNA
QKI	*ICK*	hsa-miR-607	Low miRNA
DSC3	*FGD6*	hsa-miR-105	High miRNA
PFKFB3	*ZMIZ1*	hsa-miR-185	High miRNA
POGK	*PDHA1*	hsa-miR-1	High miRNA
APH1A	*PFAS*	hsa-miR-539	Low miRNA
GRIN2A	*PCLO*	hsa-miR-203	High miRNA
ULK2	*OSBPL2*	hsa-miR-607	Low miRNA
KIAA1045	*PCLO*	hsa-miR-218	High miRNA
MECP2	*MTF1*	hsa-miR-580	Low miRNA
INCENP	*SLC7A2*	hsa-miR-424	Medium miRNA
NPR3	*TP63*	hsa-miR-340	Medium miRNA
PAICS	*UBE2G1*	hsa-miR-539	Low miRNA
FOXN3	*CTNND1*	hsa-miR-527	High miRNA
FBXL18	*SSBP3*	hsa-miR-608	Low miRNA
IQSEC3	*CNNM1*	hsa-miR-326	High miRNA
CORO2A	*RAB11FIP1*	hsa-miR-150	High miRNA
APPBP2	*YWHAZ*	hsa-miR-544	Low miRNA
PHIP	*ZC3H11A*	hsa-miR-186	High miRNA
ARHGAP5	*ATRN*	hsa-miR-607	Low miRNA
BCL2L1	*SSBP3*	hsa-miR-608	Low miRNA
TBL1XR1	*CLIP1*	hsa-miR-570	Low miRNA
KPNA4	*ANKFY1*	hsa-miR-561	Low miRNA
SNX27	*CPSF6*	hsa-miR-607	Low miRNA
PAFAH2	*FBXL18*	hsa-miR-520h	Low miRNA
TK2	*PHACTR2*	hsa-miR-552	Low miRNA
PLEKHB2	*LRPPRC*	hsa-miR-607	Low miRNA
RTF1	*ANKFY1*	hsa-miR-603	Low miRNA
ATP2B4	*ZFAND5*	hsa-miR-135a	Medium miRNA
MEF2C	*NEFL*	hsa-miR-203	High miRNA
TBL1XR1	*MTM1*	hsa-miR-570	Low miRNA
ARHGAP5	*C3orf63*	hsa-miR-607	Low miRNA
MRPL19	*KPNA1*	hsa-miR-577	Low miRNA
OCLN	*TMEM30B*	hsa-miR-607	High miRNA
ABL1	*TSC1*	hsa-miR-637	Medium miRNA

PRUNE2	*LSAMP*	hsa-miR-147	High miRNA
SENP5	*PRRG4*	hsa-miR-607	High miRNA
TSPYL1	*SV2B*	hsa-miR-485-5p	High miRNA
MIA3	*8-Sep*	hsa-miR-186	Medium miRNA
B3GAT1	*LSAMP*	hsa-miR-147	High miRNA
MBNL3	*SH2D1A*	hsa-miR-511	High miRNA
PDE4B	*ABAT*	hsa-miR-369-3p	High miRNA
SYNJ2	*EREG*	hsa-miR-586	High miRNA
SLC18A2	*VGLL3*	hsa-miR-561	High miRNA
SOSTDC1	*MPZL2*	hsa-miR-561	High miRNA
SLC6A1	*SLC1A2*	hsa-miR-27a	High miRNA
BNC2	*ITGA4*	hsa-miR-93	Low miRNA
SV2B	*ATP2B2*	hsa-miR-7	High miRNA
KLC2	*PIP4K2B*	hsa-miR-661	Low miRNA
GNA13	*MAP3K2*	hsa-miR-607	Low miRNA
KCNS1	*FBXO41*	hsa-miR-326	High miRNA
KCTD2	*HELZ*	hsa-miR-608	Low miRNA
TMED5	*YWHAZ*	hsa-miR-544	Low miRNA
MARK2	*RAB3IL1*	hsa-miR-608	Low miRNA
MAPKAPK2	*SSBP3*	hsa-miR-608	Low miRNA
PAFAH2	*KCNJ10*	hsa-miR-520h	Low miRNA
ARHGAP5	*OSBPL2*	hsa-miR-607	Low miRNA
C19orf2	*LSM14A*	hsa-miR-144	High miRNA
PACS2	*MARK2*	hsa-miR-608	Low miRNA
PELI1	*PTP4A1*	hsa-miR-607	Low miRNA
AAK1	*ARF3*	hsa-miR-329	High miRNA
KIAA0513	*KIAA0319*	hsa-miR-326	High miRNA
KCNS1	*FBXO41*	hsa-miR-149	High miRNA
ATXN1	*BBS9*	hsa-miR-340	Low miRNA
MYO1D	*WNT7B*	hsa-miR-608	High miRNA
CREB1	*CAND1*	hsa-miR-495	Low miRNA
PPP3CA	*SMCR7L*	hsa-miR-495	Low miRNA
ZNF592	*BTBD2*	hsa-miR-637	Low miRNA
BSN	*SOX4*	hsa-miR-539	Low miRNA
PPM1H	*MEF2C*	hsa-miR-570	Low miRNA
GABRA1	*RIMS3*	hsa-miR-222	High miRNA

KCNA1	*PKP1*	hsa-miR-558	Medium miRNA
NCDN	*TPPP*	hsa-miR-637	Low miRNA
CAMK2G	*CASK*	hsa-miR-495	Low miRNA
MECP2	*YWHAZ*	hsa-miR-544	Low miRNA
FGF7	*RAB3B*	hsa-miR-570	Low miRNA
OXCT1	*RFC5*	hsa-miR-340	Low miRNA
KIAA1045	*SLC8A2*	hsa-miR-625	Medium miRNA
ARHGAP5	*ATM*	hsa-miR-607	Low miRNA
SNX27	*UBE4B*	hsa-miR-607	Low miRNA
SORL1	*SLC7A11*	hsa-miR-144	High miRNA
KIAA1045	*PTPRT*	hsa-miR-218	High miRNA
SNPH	*EHD3*	hsa-miR-637	Low miRNA
MEF2C	*PREPL*	hsa-miR-410	High miRNA
RNF44	*TSC1*	hsa-miR-324-3p	Medium miRNA
GSPT1	*GNAI3*	hsa-miR-544	Low miRNA
OPA3	*STAT2*	hsa-miR-612	Low miRNA
SV2B	*SNPH*	hsa-miR-485-5p	High miRNA
ZNF592	*HIF1AN*	hsa-miR-185	High miRNA
MECP2	*USP22*	hsa-miR-646	Low miRNA
SH2D1A	*SLC38A4*	hsa-miR-607	High miRNA
KCNJ10	*MAP3K2*	hsa-miR-578	Low miRNA
SALL1	*MIA3*	hsa-miR-186	Medium miRNA
CAPRIN1	*RHOBTB3*	hsa-miR-656	Low miRNA
NRIP3	*RPH3A*	hsa-miR-485-5p	High miRNA
SENP5	*TMPRSS4*	hsa-miR-612	High miRNA
MRPS10	*TRIM13*	hsa-miR-561	Low miRNA
SLC8A2	*BSN*	hsa-miR-608	Low miRNA
KCNC1	*NRIP3*	hsa-miR-485-5p	High miRNA
ZNF532	*RRP15*	hsa-miR-200c	High miRNA
COL4A4	*CLDN18*	hsa-miR-34a	High miRNA
SNX27	*FBXW2*	hsa-miR-24	High miRNA
RAB3B	*KLF5*	hsa-miR-186	Medium miRNA
SV2B	*SLC1A2*	hsa-miR-7	High miRNA
OCLN	*NPR3*	hsa-miR-607	High miRNA
OPA3	*MAP3K2*	hsa-miR-578	Low miRNA
APPBP2	*PRPF4B*	hsa-miR-607	Low miRNA

KIAA1045	*STXBP5L*	hsa-miR-618	Low miRNA
KIAA1045	*CBX7*	hsa-miR-218	High miRNA
ANKFY1	*MAP3K2*	hsa-miR-561	Low miRNA
APPL1	*SNX19*	hsa-miR-607	Low miRNA
PHACTR2	*METTL8*	hsa-miR-552	Low miRNA
VGLL3	*SLC6A14*	hsa-miR-105	Medium miRNA
RUNX1T1	*VASH2*	hsa-miR-186	Low miRNA
FOXN3	*BMPR2*	hsa-miR-515-5p	High miRNA
MXD4	*PTPN18*	hsa-miR-34a	High miRNA
FXN	*LARP4*	hsa-miR-603	Low miRNA
SSR1	*ETF1*	hsa-miR-520h	Low miRNA
MEF2C	*SCAMP1*	hsa-miR-410	High miRNA
CRTC1	*SSBP3*	hsa-miR-608	Low miRNA
ARHGAP5	*PKD2*	hsa-miR-607	Low miRNA
CLEC2D	*TRAF3*	hsa-miR-570	Low miRNA
BSN	*GRIN2A*	hsa-miR-326	High miRNA
STXBP5L	*AAK1*	hsa-miR-539	High miRNA
QKI	*CEP170*	hsa-miR-488	Medium miRNA
WDFY3	*NIPBL*	hsa-miR-544	Low miRNA
DYNC1LI2	*TNRC6B*	hsa-miR-93	Medium miRNA
METTL8	*ZEB2*	hsa-miR-200b	High miRNA
CLCA2	*DSC2*	hsa-miR-495	Low miRNA
GPM6B	*SGCB*	hsa-miR-579	High miRNA
UBP1	*KPNA1*	hsa-miR-570	Low miRNA
RAB6B	*AAK1*	hsa-miR-329	High miRNA
UBE2J1	*LPGAT1*	hsa-miR-607	Low miRNA
RAB14	*ICK*	hsa-miR-607	Low miRNA
TMED7	*COX11*	hsa-miR-200b	High miRNA
KCNC1	*STXBP5L*	hsa-miR-605	Low miRNA
HS2ST1	*HIP1*	hsa-miR-520b	Low miRNA
KCNS1	*AAK1*	hsa-miR-149	High miRNA
ICK	*ZEB2*	hsa-miR-656	Low miRNA
BCL11B	*VASH2*	hsa-miR-20b	High miRNA
DNAJC16	*TXLNA*	hsa-miR-646	Low miRNA
PIK3R1	*TCF4*	hsa-miR-495	Medium miRNA
SSR1	*TMEM30A*	hsa-miR-656	Low miRNA

TGOLN2	*ARHGAP5*	hsa-miR-607	Low miRNA
AGPS	*PAFAH1B1*	hsa-miR-603	Low miRNA
DCP2	*YWHAZ*	hsa-miR-544	Low miRNA
CACNB4	*HLF*	hsa-miR-520g	Low miRNA
TULP4	*LPGAT1*	hsa-miR-607	Low miRNA
CTSB	*SYK*	hsa-miR-24	Medium miRNA
ARRB1	*RAB11FIP1*	hsa-miR-150	High miRNA
LARP1	*MLL*	hsa-miR-150	Medium miRNA
KIAA0513	*SCN2A*	hsa-miR-107	High miRNA
PURA	*DYNC1LI2*	hsa-miR-93	Low miRNA
SLC25A21	*GFRA1*	hsa-miR-607	Low miRNA
STYK1	*DSG3*	hsa-miR-211	Low miRNA
SLC6A14	*SLCO4C1*	hsa-miR-340	Medium miRNA
CCDC90A	*MTDH*	hsa-miR-520b	Low miRNA
RGS5	*TCF4*	hsa-miR-579	High miRNA
TULP4	*OSBPL2*	hsa-miR-607	Low miRNA
PTGFR	*RUNX1T1*	hsa-miR-384	Medium miRNA
KCNS1	*AAK1*	hsa-miR-377	High miRNA
SLC7A2	*ATP8A1*	hsa-miR-452	High miRNA
AP2A2	*PIP5K1C*	hsa-miR-661	Low miRNA
SCAMP1	*AKAP11*	hsa-miR-607	Low miRNA
ZNF264	*NUP50*	hsa-miR-603	Low miRNA
PCSK2	*PCLO*	hsa-miR-203	High miRNA
PAFAH1B1	*ANKFY1*	hsa-miR-603	Low miRNA
KCNC1	*BSN*	hsa-miR-149	High miRNA
PAK6	*AAK1*	hsa-miR-185	High miRNA
TRAF3	*DSG3*	hsa-miR-29a	Low miRNA
NCAM1	*CBX5*	hsa-miR-570	Medium miRNA
KL	*CCDC68*	hsa-miR-607	Medium miRNA
NCDN	*CACNG2*	hsa-miR-637	Low miRNA
SNX27	*ARHGAP5*	hsa-miR-607	Low miRNA
NRIP3	*DLGAP2*	hsa-miR-539	High miRNA
ARNT2	*TBC1D13*	hsa-miR-637	Medium miRNA
CANX	*RNF138*	hsa-miR-186	Medium miRNA
SCAMP4	*PIP5K1C*	hsa-miR-661	Low miRNA
ELAVL1	*RAB11FIP2*	hsa-miR-577	Low miRNA

KLC2	*SBF1*	hsa-miR-661	Low miRNA
KCNN3	*SHC3*	hsa-miR-27a	High miRNA
RAB3B	*MCTP2*	hsa-miR-607	High miRNA
AAK1	*FAIM2*	hsa-miR-7	High miRNA
PTPRN	*SCN3B*	hsa-miR-485-5p	High miRNA
COL4A4	*SLC6A14*	hsa-miR-340	Medium miRNA
ATRN	*OSBPL2*	hsa-miR-607	Low miRNA
AGPS	*RTF1*	hsa-miR-603	Low miRNA
SOSTDC1	*DSC3*	hsa-miR-561	High miRNA
RBM15B	*HGSNAT*	hsa-miR-204	Medium miRNA
AAK1	*CAMKK2*	hsa-miR-7	High miRNA
SV2B	*KHDRBS2*	hsa-miR-495	High miRNA
NCDN	*IQSEC3*	hsa-miR-608	Low miRNA
PSTPIP2	*RAB11FIP1*	hsa-miR-24	Medium miRNA
QKI	*AKAP11*	hsa-miR-142-5p	High miRNA
QKI	*PTP4A1*	hsa-miR-607	Low miRNA
GATAD2A	*ZMIZ1*	hsa-miR-661	Low miRNA
NRIP3	*RAB6B*	hsa-miR-485-5p	High miRNA
PRKAR1B	*SLC25A22*	hsa-miR-637	Low miRNA
SORL1	*SLC7A11*	hsa-miR-603	Low miRNA
UBE2J1	*ENAH*	hsa-miR-607	Low miRNA
KPNA4	*API5*	hsa-miR-607	Low miRNA
SLC8A2	*SYP*	hsa-miR-608	Low miRNA
LMO7	*CTSE*	hsa-miR-105	Medium miRNA
SSR1	*C1orf216*	hsa-miR-603	Low miRNA
ANKRD40	*LARP4*	hsa-miR-603	Low miRNA
NCAM1	*LSAMP*	hsa-miR-561	High miRNA
CCDC68	*VGLL3*	hsa-miR-142-5p	Medium miRNA
KCTD2	*HCFC1*	hsa-miR-608	Low miRNA
TMED5	*BZW1*	hsa-miR-607	Low miRNA
CUL4B	*ZNF238*	hsa-miR-561	Low miRNA
ARRB1	*UPK3B*	hsa-miR-185	Low miRNA
PPP1R16B	*FBXO41*	hsa-miR-149	High miRNA
EIF4E	*PLEKHB2*	hsa-miR-607	Low miRNA
SLC25A44	*TCF4*	hsa-miR-148a	Medium miRNA
SYNJ2BP	*ARHGAP5*	hsa-miR-607	Low miRNA

APH1A	*PJA2*	hsa-miR-539	Low miRNA
TNFSF15	*PERP*	hsa-miR-150	High miRNA
CUL4B	*KPNA4*	hsa-miR-561	Low miRNA
AAK1	*CNTNAP2*	hsa-miR-372	Low miRNA
CHML	*NPR3*	hsa-miR-607	High miRNA
CUL4B	*KPNA4*	hsa-miR-579	Low miRNA
KPNA4	*MAP3K2*	hsa-miR-561	Low miRNA
MARK2	*UBTF*	hsa-miR-608	Low miRNA
SPAST	*SCAMP1*	hsa-miR-561	Low miRNA
SLC7A8	*RAB11FIP1*	hsa-miR-185	Medium miRNA
RAP2B	*PTEN*	hsa-miR-142-5p	High miRNA
TRAF3	*MS4A1*	hsa-miR-206	Medium miRNA
POU4F1	*ONECUT2*	hsa-miR-659	High miRNA
MAP4K4	*LARP1*	hsa-miR-150	Medium miRNA
RAB22A	*CEP68*	hsa-miR-204	Medium miRNA
GABRR1	*SLCO4C1*	hsa-miR-410	Low miRNA
SSBP3	*SH3GLB2*	hsa-miR-608	Low miRNA
SV2B	*GRIN2A*	hsa-miR-136	High miRNA
TBL1XR1	*DNAJC16*	hsa-miR-570	Low miRNA
CANX	*PKD2*	hsa-miR-186	Medium miRNA
SSBP3	*GIT1*	hsa-miR-608	Low miRNA
ARHGAP5	*MAP3K2*	hsa-miR-607	Low miRNA
PBRM1	*TULP4*	hsa-miR-607	Low miRNA
APPL1	*SERINC1*	hsa-miR-607	Low miRNA
TBL1XR1	*ZFHX3*	hsa-miR-579	Low miRNA
PAK6	*CHD5*	hsa-miR-631	Low miRNA
KPNA4	*RAP2C*	hsa-miR-607	Low miRNA
IQSEC3	*CHD5*	hsa-miR-149	High miRNA
CSNK1G3	*SSR1*	hsa-miR-603	Low miRNA
RAB5A	*RBBP4*	hsa-miR-539	Low miRNA
FZR1	*MARK2*	hsa-miR-608	Low miRNA
JHDM1D	*PPP1R12B*	hsa-miR-600	Low miRNA
GCN1L1	*LARP4*	hsa-miR-607	Low miRNA
TFEC	*ENTPD1*	hsa-miR-568	Low miRNA
SNX27	*APPL1*	hsa-miR-607	Low miRNA
SORL1	*SLC7A11*	hsa-miR-607	Low miRNA

MECP2	*TEAD1*	hsa-miR-599	High miRNA
CEACAM5	*PRRG4*	hsa-miR-607	High miRNA
SSR1	*SERINC1*	hsa-miR-607	Low miRNA
ACTR8	*WNT5A*	hsa-miR-607	Low miRNA
PRRG4	*RAB11FIP1*	hsa-miR-302d	High miRNA
SYT13	*PAIP2B*	hsa-miR-203	High miRNA
EPB41L1	*FBXO41*	hsa-miR-149	High miRNA
VASH2	*GFRA1*	hsa-miR-186	Low miRNA
CREB1	*SSR1*	hsa-miR-150	Medium miRNA
SV2B	*NDRG3*	hsa-miR-485-5p	High miRNA
ATP8A2	*ST8SIA3*	hsa-miR-495	High miRNA
DOK4	*JUP*	hsa-miR-214	High miRNA
SERBP1	*C1orf216*	hsa-miR-329	Low miRNA
PIGR	*PTPRB*	hsa-miR-24	Low miRNA
ACTR8	*OSBPL2*	hsa-miR-607	Low miRNA
CRTC1	*ADORA1*	hsa-miR-24	High miRNA
SH3TC2	*RAPGEF3*	hsa-miR-484	Medium miRNA
BCL2L2	*RAPGEFL1*	hsa-miR-15a	Low miRNA
SNX27	*CTNND1*	hsa-miR-527	Medium miRNA
SYP	*CNNM1*	hsa-miR-608	Low miRNA
ATP2B3	*STXBP5L*	hsa-miR-105	Low miRNA
SLK	*UCHL5*	hsa-miR-607	High miRNA
RUFY3	*SEMA5A*	hsa-miR-186	Medium miRNA
SCN3B	*CAMK2A*	hsa-miR-485-5p	High miRNA
SLC7A2	*ATP8A1*	hsa-miR-495	Low miRNA
PCM1	*GOSR1*	hsa-miR-150	Medium miRNA
PEX13	*ABI2*	hsa-miR-302d	Low miRNA
SV2B	*CAMK2G*	hsa-miR-495	High miRNA
CCDC68	*ADAMTSL3*	hsa-miR-340	Medium miRNA
CSNK1G3	*CPSF6*	hsa-miR-603	Low miRNA
BSN	*NMNAT2*	hsa-miR-185	High miRNA
NR3C1	*ARHGEF3*	hsa-miR-181a	Low miRNA
CLDN1	*SFTPB*	hsa-miR-600	High miRNA
SNX27	*CBFB*	hsa-miR-607	Low miRNA
PRPF4B	*SMARCA5*	hsa-miR-606	Low miRNA
POU2F1	*ASB1*	hsa-miR-372	Low miRNA

SH3TC2	*DLG2*	hsa-miR-185	High miRNA
MECP2	*ZC3H11A*	hsa-miR-186	High miRNA
MRPL19	*PEX19*	hsa-miR-539	Low miRNA
UBE4B	*CBFB*	hsa-miR-607	Low miRNA
GRIN2A	*KIAA0319*	hsa-miR-326	High miRNA
POLR2D	*CNOT8*	hsa-miR-607	Low miRNA
GREM2	*DLGAP2*	hsa-miR-205	Low miRNA
CLEC2D	*RHOH*	hsa-miR-570	Low miRNA
EPHB2	*UCK2*	hsa-miR-593	Medium miRNA
GFRA1	*AGTR2*	hsa-miR-607	Low miRNA
SORBS3	*UBTF*	hsa-miR-608	Low miRNA
PDLIM5	*OSBPL2*	hsa-miR-607	Low miRNA
PDE4D	*SLC7A2*	hsa-miR-495	Medium miRNA
THSD4	*EHF*	hsa-miR-607	High miRNA
POU2AF1	*IRF4*	hsa-miR-203	Low miRNA
HEG1	*GOSR1*	hsa-miR-106b	Medium miRNA
ATRN	*ASB1*	hsa-miR-607	Low miRNA
SSR1	*ABI2*	hsa-miR-150	Medium miRNA
TNS1	*GOSR1*	hsa-miR-326	Low miRNA
AP2B1	*MAPRE2*	hsa-miR-583	Low miRNA
HELZ	*ASB1*	hsa-miR-607	Low miRNA
YTHDC1	*OSBPL2*	hsa-miR-607	Low miRNA
FZR1	*HCFC1*	hsa-miR-608	Low miRNA
SLC8A2	*GRIN2A*	hsa-miR-7	High miRNA
C17orf63	*DVL3*	hsa-miR-625	Low miRNA
NCDN	*PRKAR1B*	hsa-miR-637	Low miRNA
SH3PXD2A	*CHD5*	hsa-miR-149	High miRNA
PAFAH1B1	*RTF1*	hsa-miR-603	Low miRNA
SNX19	*OSBPL2*	hsa-miR-607	Low miRNA
CACNG2	*KIAA1045*	hsa-miR-637	Low miRNA
RANBP2	*ARHGAP5*	hsa-miR-607	Low miRNA
ZEB1	*DOCK4*	hsa-miR-655	High miRNA
RAP2B	*TBC1D4*	hsa-miR-142-5p	High miRNA
AGPS	*CYP20A1*	hsa-miR-656	Low miRNA
NHLH2	*ONECUT2*	hsa-miR-561	Medium miRNA
CUL4B	*EXOC5*	hsa-miR-583	Low miRNA

MYO1D	*CLMN*	hsa-miR-608	Low miRNA
ULK2	*C18orf1*	hsa-miR-607	Low miRNA
QKI	*GPM6B*	hsa-miR-579	High miRNA
ULK2	*PAIP2B*	hsa-miR-515-5p	Low miRNA
KIAA0408	*DLG3*	hsa-miR-646	Low miRNA
QKI	*TCF4*	hsa-miR-203	High miRNA
TPM3	*CAMK2A*	hsa-miR-485-5p	High miRNA
SUZ12	*TNPO1*	hsa-miR-607	High miRNA
HEG1	*EIF2C1*	hsa-miR-106b	Medium miRNA
PDLIM5	*ASB1*	hsa-miR-372	Low miRNA
PPM1H	*AAK1*	hsa-miR-214	Low miRNA
ARRB1	*SFTPB*	hsa-miR-608	Low miRNA
AGPS	*KPNA4*	hsa-miR-579	Low miRNA
DSC3	*SLC12A6*	hsa-miR-203	Medium miRNA
GCN1L1	*NRIP1*	hsa-miR-607	Low miRNA
MTMR9	*PBRM1*	hsa-miR-607	Low miRNA
OPA1	*OSBPL2*	hsa-miR-607	Low miRNA
SSR1	*CCPG1*	hsa-miR-545	Low miRNA
NCDN	*BSN*	hsa-miR-608	Low miRNA
THRAP3	*BRAP*	hsa-miR-579	Low miRNA
DDX21	*TRAM2*	hsa-miR-31	High miRNA
ATP11B	*RAP2B*	hsa-miR-186	Medium miRNA
FOXN2	*SP4*	hsa-miR-381	Medium miRNA
ZBTB6	*THRAP3*	hsa-miR-561	Low miRNA
OPA3	*MAP3K2*	hsa-miR-302b	Low miRNA
ARHGAP5	*WIPF2*	hsa-miR-607	Low miRNA
AGPS	*GOSR2*	hsa-miR-603	Low miRNA
BCL2L2	*GABARAPL1*	hsa-miR-15b	Low miRNA
WDFY3	*LIMCH1*	hsa-miR-620	Low miRNA
YWHAZ	*NUDT4*	hsa-miR-544	Low miRNA
SEC63	*CSNK1A1*	hsa-miR-186	Medium miRNA
DISC1	*SEMA5A*	hsa-miR-203	High miRNA
C11orf41	*KIF3A*	hsa-miR-335	High miRNA
TRPS1	*TTC28*	hsa-miR-204	Medium miRNA
YTHDC1	*KIAA0226*	hsa-miR-607	Low miRNA
PAK6	*FBXO41*	hsa-miR-185	High miRNA

USP46	*PBLD*	hsa-miR-519d	Low miRNA
PRG4	*GABRR1*	hsa-miR-410	High miRNA
TSC1	*HGSNAT*	hsa-miR-204	Medium miRNA
KCNC1	*KIAA0319*	hsa-miR-326	High miRNA
APPBP2	*C3orf63*	hsa-miR-607	Low miRNA
SSBP3	*RAB3IL1*	hsa-miR-608	Low miRNA
PPP1R12B	*HIF1AN*	hsa-miR-372	Low miRNA
TBL1XR1	*PAFAH1B1*	hsa-miR-16	Medium miRNA
SH3TC2	*AAK1*	hsa-miR-30b	Medium miRNA
APPL1	*PRPF4B*	hsa-miR-607	Low miRNA
PDE4B	*FZD3*	hsa-miR-369-3p	High miRNA
MEF2C	*TRIM23*	hsa-miR-410	High miRNA
CNTNAP2	*CBX7*	hsa-miR-218	High miRNA
GRIN2A	*CNNM1*	hsa-miR-326	High miRNA
ZNF24	*YTHDC1*	hsa-miR-607	Low miRNA
LARP4	*NRIP1*	hsa-miR-607	Low miRNA
PTPRB	*VGLL3*	hsa-miR-579	Low miRNA
FMO2	*OCLN*	hsa-miR-340	Medium miRNA
GGCX	*BZW1*	hsa-miR-607	Low miRNA
TPPP	*SEC14L5*	hsa-miR-661	Low miRNA
CFLAR	*LARP4*	hsa-miR-603	Low miRNA
FAM134A	*GLG1*	hsa-miR-650	High miRNA
TAP2	*GCNT2*	hsa-miR-136	Medium miRNA
FUT2	*POU2AF1*	hsa-miR-485-5p	Low miRNA
CACNG2	*IQSEC3*	hsa-miR-637	Low miRNA
MRPL19	*YWHAZ*	hsa-miR-544	Low miRNA
C12orf5	*SELT*	hsa-miR-520g	Low miRNA
CCNJ	*DHTKD1*	hsa-miR-302d	Low miRNA
OPA3	*MAP3K2*	hsa-miR-302c	Low miRNA
ADIPOR2	*FOXK2*	hsa-miR-384	Low miRNA
GPR107	*RNF14*	hsa-miR-587	Low miRNA
CSNK1A1	*PKD2*	hsa-miR-186	Medium miRNA
LPGAT1	*NRIP1*	hsa-miR-656	Low miRNA
SLC12A5	*IQSEC3*	hsa-miR-663	Low miRNA
QKI	*TCF4*	hsa-miR-579	High miRNA
STYK1	*CACNG2*	hsa-miR-204	Medium miRNA

KRAS	*PTCD3*	hsa-miR-217	Medium miRNA
EFHD2	*SSBP3*	hsa-miR-608	Low miRNA
IPO9	*KIAA0226*	hsa-miR-372	High miRNA
SENP5	*KLK10*	hsa-miR-612	High miRNA
TBL1XR1	*KPNA4*	hsa-miR-579	Low miRNA
FMO2	*LMO7*	hsa-miR-340	Medium miRNA
DDX21	*LPP*	hsa-miR-31	High miRNA
TK2	*SLC7A8*	hsa-miR-766	Low miRNA
AP1G1	*GFPT1*	hsa-miR-520h	Low miRNA
CPEB3	*MEF2C*	hsa-miR-570	Low miRNA
TACC1	*USP9X*	hsa-miR-570	High miRNA
FBXO41	*AAK1*	hsa-miR-302d	Low miRNA
KIAA1045	*GRIN2A*	hsa-miR-593	Low miRNA
AAK1	*CNNM1*	hsa-miR-326	High miRNA
C16orf58	*PIP4K2B*	hsa-miR-661	Low miRNA
PRSS16	*PERP*	hsa-miR-539	High miRNA
C1orf216	*LARP4*	hsa-miR-603	Low miRNA
KPNA4	*EXOC5*	hsa-miR-410	High miRNA
PRKCA	*TNS1*	hsa-miR-326	Low miRNA
DCP2	*NDFIP1*	hsa-miR-105	Low miRNA
PRKCI	*ABCE1*	hsa-miR-656	Low miRNA
ST8SIA3	*ONECUT2*	hsa-miR-561	High miRNA
SNX27	*ZMIZ1*	hsa-miR-612	Medium miRNA
YTHDC1	*ATRN*	hsa-miR-607	Low miRNA
YME1L1	*MCTS1*	hsa-miR-411	Low miRNA
GNG13	*AAK1*	hsa-miR-600	Low miRNA
POU2AF1	*IL21R*	hsa-miR-766	Low miRNA
FBXO41	*AAK1*	hsa-miR-302a	Low miRNA
CDC42BPB	*PIP5K1C*	hsa-miR-608	Low miRNA
SORL1	*ARHGAP5*	hsa-miR-607	Low miRNA
KIF1B	*NCAN*	hsa-miR-599	High miRNA
CCDC68	*SLCO4C1*	hsa-miR-340	Medium miRNA
PGR	*THSD4*	hsa-miR-607	High miRNA
APPL1	*APPBP2*	hsa-miR-607	Low miRNA
HEG1	*EIF2C1*	hsa-miR-106a	Low miRNA
C6orf120	*COX11*	hsa-miR-200b	High miRNA

STXBP5L	*GRIN2A*	hsa-miR-618	Low miRNA
JHDM1D	*ZEB2*	hsa-miR-656	Low miRNA
KHDRBS2	*CAMK2G*	hsa-miR-495	High miRNA
JHDM1D	*ZEB2*	hsa-miR-561	Low miRNA
CEP68	*HGSNAT*	hsa-miR-204	Medium miRNA
SV2B	*STXBP5L*	hsa-miR-758	High miRNA
IPO9	*GOSR1*	hsa-miR-150	Medium miRNA
AAK1	*CHD5*	hsa-miR-149	High miRNA
GCN1L1	*TMF1*	hsa-miR-607	Low miRNA
FBXO41	*VIPR1*	hsa-miR-138	High miRNA
NMNAT2	*ULK2*	hsa-miR-185	High miRNA
SS18	*TOLLIP*	hsa-miR-144	Low miRNA
RNF216	*ARFRP1*	hsa-miR-661	Low miRNA
UTP14C	*TRIM33*	hsa-miR-586	Low miRNA
SH3TC2	*TPPP*	hsa-miR-484	Medium miRNA
PTGFR	*SGCD*	hsa-miR-384	High miRNA
PRKCI	*NRIP1*	hsa-miR-656	Low miRNA
NBR1	*HGSNAT*	hsa-miR-204	Medium miRNA
CAB39	*ACTR2*	hsa-miR-495	Low miRNA
EPB49	*IQSEC3*	hsa-miR-637	Low miRNA
RTF1	*SSR1*	hsa-miR-603	Low miRNA
NEFL	*AAK1*	hsa-miR-203	High miRNA
FAM105A	*ENTPD1*	hsa-miR-641	Low miRNA
GABRA1	*TRHDE*	hsa-miR-580	Low miRNA
YTHDC1	*ASB1*	hsa-miR-607	Low miRNA
BSN	*CNNM1*	hsa-miR-608	Low miRNA
TMED5	*GSPT1*	hsa-miR-544	Low miRNA
SSR3	*KPNA4*	hsa-miR-570	Low miRNA
UBE4B	*SNX19*	hsa-miR-607	Low miRNA
SORBS3	*MARK2*	hsa-miR-608	Low miRNA
NUDT4	*DGAT1*	hsa-miR-661	Low miRNA
VPS37C	*MAFG*	hsa-miR-29a	High miRNA
SMARCC2	*MLL*	hsa-miR-150	Medium miRNA
PTPRB	*FMO2*	hsa-miR-607	Medium miRNA
C3orf58	*ABCA5*	hsa-miR-559	Medium miRNA
SSR1	*RNF38*	hsa-miR-603	Low miRNA

ENAH	*SNX27*	hsa-miR-607	Low miRNA
TACC1	*USP9X*	hsa-miR-134	Low miRNA
SEC63	*PPP3CA*	hsa-miR-495	Low miRNA
MME	*MCTP2*	hsa-miR-561	Medium miRNA
BSN	*AAK1*	hsa-miR-539	High miRNA
MARK2	*CRTC1*	hsa-miR-608	Low miRNA
PRKCH	*C18orf1*	hsa-miR-578	Low miRNA
NPAS3	*CALM1*	hsa-miR-579	High miRNA
TK2	*SLC7A11*	hsa-miR-522	Low miRNA
FXR1	*WDR82*	hsa-miR-607	Low miRNA
SH3TC2	*ADARB2*	hsa-miR-769-5p	High miRNA
ZFHX3	*MAP3K2*	hsa-miR-579	Low miRNA
QKI	*FAM70A*	hsa-miR-200a	Medium miRNA
SEC63	*TMEM30A*	hsa-miR-656	Low miRNA
AAK1	*ADARB2*	hsa-miR-484	Medium miRNA
IQCE	*ARFRP1*	hsa-miR-661	Low miRNA
MNT	*MGRN1*	hsa-miR-608	Medium miRNA
TBC1D12	*LPGAT1*	hsa-miR-607	Low miRNA
BSN	*EPB41L1*	hsa-miR-149	High miRNA
HIC2	*MAPT*	hsa-miR-637	Low miRNA
FZR1	*OPA3*	hsa-miR-608	Low miRNA
ZER1	*FBXL18*	hsa-miR-661	Low miRNA
CBFA2T2	*KCNN3*	hsa-miR-24	High miRNA
CAMK2A	*KIAA0319*	hsa-miR-326	High miRNA
SPAG9	*CAPRIN1*	hsa-miR-656	Low miRNA
TOMM70A	*APPBP2*	hsa-miR-607	Low miRNA
TK2	*GOSR1*	hsa-miR-326	Low miRNA
CUL4B	*MAP3K2*	hsa-miR-579	Low miRNA
RNF125	*IL17RA*	hsa-miR-612	Low miRNA
FMO2	*SLC6A14*	hsa-miR-340	Medium miRNA
DCP2	*DCUN1D1*	hsa-miR-561	Low miRNA
ATP6V0A1	*TRIM3*	hsa-miR-608	Low miRNA
CLCA2	*TMEM30B*	hsa-miR-495	Low miRNA
QKI	*TOMM70A*	hsa-miR-607	Low miRNA
ZEB1	*QKI*	hsa-miR-203	High miRNA
TM9SF3	*PKD2*	hsa-miR-607	Low miRNA

MME	*PTGFR*	hsa-miR-539	High miRNA
PRPF40A	*RAB11FIP2*	hsa-miR-410	Low miRNA
PAX8	*RAPGEF3*	hsa-miR-324-3p	Low miRNA
DDX21	*CHST3*	hsa-miR-607	Low miRNA
KIAA0513	*MAST3*	hsa-miR-766	Medium miRNA
TBL1XR1	*TJP1*	hsa-miR-105	Low miRNA
SCN3B	*RAB6B*	hsa-miR-485-5p	High miRNA
POLI	*S100PBP*	hsa-miR-583	Low miRNA
FGF2	*MAP3K2*	hsa-miR-369-3p	Low miRNA
UBE2J1	*MRPL19*	hsa-miR-633	Low miRNA
SEC63	*SMARCA5*	hsa-miR-186	Medium miRNA
ZEB1	*KIF3A*	hsa-miR-203	High miRNA
SLC12A6	*DSG3*	hsa-miR-211	Low miRNA
CLEC2D	*STX11*	hsa-miR-568	Medium miRNA
SNX27	*OPA1*	hsa-miR-607	Low miRNA
FZD3	*ASB1*	hsa-miR-372	Low miRNA
TJP1	*SS18*	hsa-miR-144	Low miRNA
FRS2	*MAP3K2*	hsa-miR-96	Low miRNA
PURA	*HIPK2*	hsa-miR-15b	Low miRNA
ARRB1	*AOC3*	hsa-miR-150	High miRNA
PPIL2	*POLH*	hsa-miR-661	Low miRNA
TNFSF15	*STEAP4*	hsa-miR-369-3p	Medium miRNA
LASP1	*LARP4*	hsa-miR-603	Low miRNA
PTP4A1	*FAM110B*	hsa-miR-607	Low miRNA
PPM1H	*TULP4*	hsa-miR-607	Low miRNA
ZFHX3	*THRAP3*	hsa-miR-579	Low miRNA
ZBTB6	*ZNF238*	hsa-miR-561	Low miRNA
SH3TC2	*LDB3*	hsa-miR-607	Low miRNA
THRAP3	*MAP3K2*	hsa-miR-579	Low miRNA
SEC16A	*EIF2C1*	hsa-miR-519a	Low miRNA
C17orf63	*TSC1*	hsa-miR-625	Low miRNA
TNPO1	*HNRNPA0*	hsa-miR-186	Medium miRNA
SLC17A6	*SV2B*	hsa-miR-495	High miRNA
TRAF1	*TRAF3*	hsa-miR-214	Low miRNA
ELAVL1	*KPNA1*	hsa-miR-577	Low miRNA
SIRPA	*ADORA1*	hsa-miR-24	High miRNA

SPTBN1	*KIF3A*	hsa-miR-203	High miRNA
RAPGEF3	*ADARB2*	hsa-miR-484	Medium miRNA
ENAH	*PREPL*	hsa-miR-607	Low miRNA
CSNK1A1	*ZNF148*	hsa-miR-186	Medium miRNA
TOM1L2	*FBXL18*	hsa-miR-650	Medium miRNA
BSN	*KCNS1*	hsa-miR-326	High miRNA
ABI2	*TOLLIP*	hsa-miR-144	Low miRNA
PAFAH1B1	*PRPF4B*	hsa-miR-607	Low miRNA
CBX5	*PPP3CA*	hsa-miR-381	Low miRNA
CNTN2	*SH3TC2*	hsa-miR-573	Low miRNA
TSPYL1	*AAK1*	hsa-miR-485-5p	High miRNA
LPHN1	*KIAA0513*	hsa-miR-623	Low miRNA
RAB11A	*TM9SF3*	hsa-miR-579	Low miRNA
KPNA4	*PRKAR1A*	hsa-miR-561	Low miRNA
IPO9	*TRPS1*	hsa-miR-372	High miRNA
EIF2C1	*ZNF654*	hsa-miR-578	Low miRNA
ZMYM2	*TNRC6B*	hsa-miR-181b	High miRNA
CACNG2	*STXBP5L*	hsa-miR-204	Medium miRNA
SLC22A3	*SDPR*	hsa-miR-186	Low miRNA
AGPS	*CSNK1G3*	hsa-miR-603	Low miRNA
MCTS1	*SELT*	hsa-miR-520g	Low miRNA
SLC7A11	*FUT9*	hsa-miR-181d	Low miRNA
NOTCH2	*CEP68*	hsa-miR-204	Medium miRNA
RAB3B	*DSG3*	hsa-miR-607	High miRNA
ICK	*TTC33*	hsa-miR-656	Low miRNA
NCDN	*PDE1B*	hsa-miR-608	Low miRNA
QKI	*KAL1*	hsa-miR-340	Low miRNA
DHTKD1	*HIF1AN*	hsa-miR-302d	Low miRNA
KIAA1324	*CYB561*	hsa-miR-661	Medium miRNA
C1orf21	*NMNAT2*	hsa-miR-185	High miRNA
PPP1R2	*SMAD2*	hsa-miR-410	Low miRNA
CCDC68	*EMCN*	hsa-miR-340	Medium miRNA
CEACAM5	*ATP8A1*	hsa-miR-607	High miRNA
KHDRBS2	*AAK1*	hsa-miR-495	High miRNA
GPM6A	*STX12*	hsa-miR-607	High miRNA
WIPF1	*GFPT1*	hsa-miR-429	Low miRNA

PCLO	*CACNB4*	hsa-miR-218	High miRNA
ASB1	*CPSF6*	hsa-miR-607	Low miRNA
NOS1AP	*GRIN2A*	hsa-miR-136	High miRNA
YWHAZ	*EIF1AX*	hsa-miR-544	Low miRNA
SLK	*CLCN4*	hsa-miR-607	Low miRNA
SH3BP2	*CRTC1*	hsa-miR-24	High miRNA
GPM6B	*PMP2*	hsa-miR-579	High miRNA
SYT13	*FBXO41*	hsa-miR-205	Low miRNA
NRP2	*PAPPA*	hsa-miR-15a	Medium miRNA
NCDN	*SH3GLB2*	hsa-miR-608	Low miRNA
TRPS1	*ATXN1*	hsa-miR-204	Medium miRNA
TRIM9	*LARP1*	hsa-miR-150	Medium miRNA
SV2B	*DLGAP2*	hsa-miR-539	High miRNA
SELE	*ARRB1*	hsa-miR-200a	High miRNA
OSBPL2	*WIPF2*	hsa-miR-607	Low miRNA
LSAMP	*APC*	hsa-miR-561	High miRNA
SLC8A2	*IQSEC3*	hsa-miR-608	Low miRNA
FOXJ3	*RORA*	hsa-miR-106a	Low miRNA
SLC12A5	*FBXW7*	hsa-miR-25	Low miRNA
PHKA1	*RORA*	hsa-miR-520e	Low miRNA
SH3TC2	*SH3PXD2A*	hsa-miR-515-5p	Low miRNA
OSBPL8	*PYROXD1*	hsa-miR-186	Low miRNA
HTR2C	*PCLO*	hsa-miR-203	High miRNA
RAC2	*IKZF1*	hsa-miR-608	Low miRNA
CNKSR2	*RIMBP2*	hsa-miR-30d	Medium miRNA
PRRG1	*SPTBN1*	hsa-miR-616	Low miRNA
SH3TC2	*CLMN*	hsa-miR-181b	High miRNA
AAK1	*PPP3CA*	hsa-miR-495	High miRNA
TRPS1	*SLC7A11*	hsa-miR-579	Low miRNA
APPBP2	*TCF4*	hsa-miR-203	Medium miRNA
GALNT4	*EHF*	hsa-miR-181c	Low miRNA
NAP1L1	*PPP1R12B*	hsa-miR-511	Low miRNA
ZNF592	*RANBP3*	hsa-miR-637	Low miRNA
ARL5A	*COX11*	hsa-miR-200b	High miRNA
PAFAH1B1	*GOSR2*	hsa-miR-603	Low miRNA
FPGT	*SSR1*	hsa-miR-603	Low miRNA

SH3TC2	*LHPP*	hsa-miR-608	Low miRNA
LARP4	*MAP3K2*	hsa-miR-559	Low miRNA
SOX2	*TCF4*	hsa-miR-557	Low miRNA
GCN1L1	*UBP1*	hsa-miR-607	Low miRNA
LPGAT1	*DNM3*	hsa-miR-607	Low miRNA
ASTN1	*LSAMP*	hsa-miR-544	High miRNA
KIF5C	*ARHGEF6*	hsa-miR-559	High miRNA
ABR	*DOCK3*	hsa-miR-125b	High miRNA
TBL1XR1	*RNF38*	hsa-miR-367	Low miRNA
TARDBP	*ATRN*	hsa-miR-607	Low miRNA
CTSB	*EMR2*	hsa-miR-150	Low miRNA
ZFHX3	*MTM1*	hsa-miR-570	Low miRNA
LSAMP	*SEMA6A*	hsa-miR-147	High miRNA
EIF1AX	*ATRN*	hsa-miR-544	Low miRNA
SV2B	*KIAA0319*	hsa-miR-23a	Low miRNA
PRKACB	*GSPT2*	hsa-miR-607	High miRNA
SSR1	*CBFB*	hsa-miR-802	Low miRNA
FBXO41	*CNNM1*	hsa-miR-326	High miRNA
TNPO1	*GOSR1*	hsa-miR-186	Medium miRNA
GNA13	*FRS2*	hsa-miR-96	Low miRNA
SYT13	*SV2B*	hsa-miR-527	Medium miRNA
PPM1H	*PIP5K1C*	hsa-miR-661	Low miRNA
AP1S2	*QKI*	hsa-miR-561	High miRNA
FGF7	*RAB3B*	hsa-miR-607	High miRNA
SSR1	*LASP1*	hsa-miR-603	Low miRNA
ARRB1	*KCNJ12*	hsa-miR-125b	High miRNA
GCN1L1	*PKD2*	hsa-miR-607	Low miRNA
PPM1H	*HLF*	hsa-miR-570	Low miRNA
JHDM1D	*ACVR2B*	hsa-miR-369-3p	Medium miRNA
BSN	*AAK1*	hsa-miR-326	High miRNA
FBXO41	*SEC14L5*	hsa-miR-138	High miRNA
TMED5	*SSR1*	hsa-miR-607	Low miRNA
THSD4	*KCNJ13*	hsa-miR-607	Medium miRNA
YWHAZ	*USP9X*	hsa-miR-544	Low miRNA
AQP4	*SLC7A11*	hsa-miR-607	Low miRNA
UBE2J1	*SNX27*	hsa-miR-607	Low miRNA

AZIN1	*PFN2*	hsa-miR-520h	Low miRNA
PDE1B	*CNNM1*	hsa-miR-608	Low miRNA
MIA3	*HECA*	hsa-miR-607	Low miRNA
RAB14	*ZMPSTE24*	hsa-miR-577	Low miRNA
EIF4E	*CNOT8*	hsa-miR-607	Low miRNA
TRPS1	*FAM125B*	hsa-miR-204	High miRNA
EXOC5	*ANKFY1*	hsa-miR-186	Low miRNA
CAMTA1	*RIMBP2*	hsa-miR-106a	Low miRNA
OPA3	*PGPEP1*	hsa-miR-372	Low miRNA
APPBP2	*FMR1*	hsa-miR-607	Low miRNA
SSR1	*CDS2*	hsa-miR-603	Low miRNA
C21orf91	*DSG3*	hsa-miR-655	High miRNA
DCAKD	*FBXL18*	hsa-miR-661	Low miRNA
ELAVL1	*ABI2*	hsa-miR-522	Low miRNA
PCSK2	*PTPRT*	hsa-miR-203	High miRNA
ARNT2	*B3GAT1*	hsa-miR-147	High miRNA
ENAH	*PAICS*	hsa-miR-485-5p	Low miRNA
APPBP2	*LRPPRC*	hsa-miR-607	Low miRNA
MBNL3	*CLEC2D*	hsa-miR-587	Low miRNA
AQP2	*NKX2-8*	hsa-miR-637	Medium miRNA
KIAA1045	*AAK1*	hsa-miR-326	High miRNA
PAFAH1B1	*CPSF6*	hsa-miR-607	Low miRNA
ERBB2IP	*LSAMP*	hsa-miR-144	Medium miRNA
NFATC2IP	*HIP1*	hsa-miR-520b	Low miRNA
ACTR8	*IREB2*	hsa-miR-607	Low miRNA
TGOLN2	*PKD2*	hsa-miR-607	Low miRNA
FGF7	*SLC6A14*	hsa-miR-340	Medium miRNA
PDCD6IP	*ZFAND5*	hsa-miR-607	Low miRNA
HOOK1	*ONECUT2*	hsa-miR-409-3p	High miRNA
KIF1B	*SLC1A4*	hsa-miR-545	Medium miRNA
TBL1XR1	*FGF2*	hsa-miR-570	Low miRNA
DCUN1D1	*PRKD3*	hsa-miR-340	Low miRNA
NCDN	*CACNG2*	hsa-miR-608	Low miRNA
PPM1H	*SERINC1*	hsa-miR-607	Low miRNA
BSN	*IQSEC3*	hsa-miR-608	Low miRNA
PAK6	*RAB11FIP1*	hsa-miR-185	Medium miRNA

ARNT2	*PMP2*	hsa-miR-23a	Medium miRNA
CUL4B	*SSR1*	hsa-miR-570	Low miRNA
KCNC1	*PTPRN*	hsa-miR-485-5p	High miRNA
GDPD5	*POLH*	hsa-miR-588	Low miRNA
SCN3B	*AAK1*	hsa-miR-485-5p	High miRNA
MIA3	*GRIA3*	hsa-miR-186	Medium miRNA
ZNF24	*ICK*	hsa-miR-607	Low miRNA
PITPNA	*CFLAR*	hsa-miR-552	Low miRNA
CEACAM5	*LGALS8*	hsa-miR-607	High miRNA
ZNF264	*ASB1*	hsa-miR-214	High miRNA
TK2	*TBC1D13*	hsa-miR-326	Low miRNA
AKAP5	*ATP8A1*	hsa-miR-607	Medium miRNA
GTF2H5	*SELT*	hsa-miR-520g	Low miRNA
GSPT1	*C12orf5*	hsa-miR-603	Low miRNA
ANKFY1	*FBXL18*	hsa-miR-326	Low miRNA
TMEM127	*CEP68*	hsa-miR-204	Medium miRNA
ADORA1	*KIF1C*	hsa-miR-24	High miRNA
TPPP	*SEC14L5*	hsa-miR-370	High miRNA
PAX8	*RAPGEF3*	hsa-miR-326	Medium miRNA
CCRL1	*ADAMTSL3*	hsa-miR-340	Medium miRNA
MTMR9	*ICK*	hsa-miR-607	Low miRNA
FAM46C	*LAMP3*	hsa-miR-93	High miRNA
TRAF3	*MS4A1*	hsa-miR-1	Low miRNA
RAB6B	*KIAA0513*	hsa-miR-485-5p	High miRNA
PRDM2	*LANCL1*	hsa-miR-211	High miRNA
ARNT2	*LSAMP*	hsa-miR-147	High miRNA
AP1S2	*RAP2A*	hsa-miR-561	High miRNA
SH3TC2	*ADARB2*	hsa-miR-484	Medium miRNA
SLC16A2	*FBXW2*	hsa-miR-24	High miRNA
RAB5A	*APH1A*	hsa-miR-539	Low miRNA
FOXRED2	*KIF1C*	hsa-miR-552	Low miRNA
POU3F2	*PMP2*	hsa-miR-579	High miRNA
NFATC2IP	*HIP1*	hsa-miR-520e	Low miRNA
SMAD2	*KPNA1*	hsa-miR-577	Low miRNA
ZCCHC14	*TCF4*	hsa-miR-141	High miRNA
QKI	*TRIM2*	hsa-miR-488	Low miRNA

SV2B	*DNAJC6*	hsa-miR-23a	Low miRNA
KCNK15	*PTPN3*	hsa-miR-497	Medium miRNA
PPP1R16B	*BSN*	hsa-miR-149	High miRNA
ZNF532	*USP46*	hsa-miR-200c	High miRNA
RAB22A	*CSNK1A1*	hsa-miR-186	Medium miRNA
SLC35A5	*CAPRIN1*	hsa-miR-656	Low miRNA
QKI	*FNDC3A*	hsa-miR-561	Low miRNA
PJA2	*SGCB*	hsa-miR-579	High miRNA
PDLIM5	*FZD3*	hsa-miR-520e	High miRNA
MYB	*RAB11FIP1*	hsa-miR-150	High miRNA
SSR1	*SENP6*	hsa-miR-802	Low miRNA
MECP2	*PIP5K1C*	hsa-miR-661	Low miRNA
PPM1H	*PHLDB1*	hsa-miR-661	Low miRNA
APPBP2	*LPGAT1*	hsa-miR-607	Low miRNA
ZNF264	*HEG1*	hsa-miR-372	Low miRNA
KIF5C	*SEMA5A*	hsa-miR-186	Medium miRNA
NOS1AP	*AAK1*	hsa-miR-136	High miRNA
APPL1	*OSBPL2*	hsa-miR-607	Low miRNA
MAP3K3	*PPM1H*	hsa-miR-661	Low miRNA
FAM134C	*DVL3*	hsa-miR-485-5p	Low miRNA
PACS2	*RAB3IL1*	hsa-miR-608	Low miRNA
VASH1	*NOTCH2*	hsa-miR-204	High miRNA
TANC2	*ZNF654*	hsa-miR-578	Low miRNA
POLH	*RAB5B*	hsa-miR-661	Low miRNA
KCNS1	*KIAA0513*	hsa-miR-326	High miRNA
RAB14	*ASB1*	hsa-miR-607	Low miRNA
ZNF148	*GOLGA1*	hsa-miR-522	Low miRNA
ARHGAP5	*ZNF148*	hsa-miR-607	Low miRNA
OXR1	*ITGAV*	hsa-miR-579	High miRNA
GCN1L1	*RANBP2*	hsa-miR-607	Low miRNA
FBXO41	*AAK1*	hsa-miR-326	High miRNA
USP46	*TRIM33*	hsa-miR-200c	High miRNA
RAB7L1	*HIP1*	hsa-miR-302d	Low miRNA
PIP4K2B	*ARFRP1*	hsa-miR-661	Low miRNA
FUT1	*EPN3*	hsa-miR-661	Medium miRNA
EIF2C1	*TSC1*	hsa-miR-637	Low miRNA

RAB11FIP2	*CYP20A1*	hsa-miR-577	Low miRNA
SV2B	*ATP8A2*	hsa-miR-495	High miRNA
PFAS	*ELAVL1*	hsa-miR-539	Low miRNA
NT5DC3	*CRTC1*	hsa-miR-612	Low miRNA
TULP4	*TRAM2*	hsa-miR-539	Low miRNA
ZNF592	*HDAC5*	hsa-miR-185	High miRNA
PPM1H	*PPP1R16B*	hsa-miR-661	Low miRNA
TMED7	*EIF4E*	hsa-miR-607	Low miRNA
C1orf21	*EIF4B*	hsa-miR-150	Medium miRNA
ATXN1	*CEP68*	hsa-miR-204	Medium miRNA
PTGFR	*RUNX1T1*	hsa-miR-495	High miRNA
NPTXR	*NPTX1*	hsa-miR-152	Low miRNA
RAP2A	*ICK*	hsa-miR-568	Medium miRNA
ALG9	*DCTN5*	hsa-miR-612	Low miRNA
CAND1	*PTCD3*	hsa-miR-495	Low miRNA
DLGAP2	*RAPGEFL1*	hsa-miR-15b	Low miRNA
CUL4B	*TBL1XR1*	hsa-miR-579	Low miRNA
TK2	*CFLAR*	hsa-miR-552	Low miRNA
TFE3	*TBC1D2B*	hsa-miR-608	Low miRNA
TMEM57	*KIDINS220*	hsa-miR-141	High miRNA
HS2ST1	*PHACTR4*	hsa-miR-520b	Low miRNA
HECA	*DVL3*	hsa-miR-559	Low miRNA
MYT1L	*AAK1*	hsa-miR-495	High miRNA
SEMA5A	*GRIA3*	hsa-miR-186	Medium miRNA
SLC39A9	*APOOL*	hsa-miR-539	Medium miRNA
LRPPRC	*PAICS*	hsa-miR-485-5p	Medium miRNA
MAP3K3	*RAB5B*	hsa-miR-661	Low miRNA
PTPN14	*PAPPA*	hsa-miR-497	Low miRNA
SLC9A6	*TCF4*	hsa-miR-142-5p	High miRNA
AGPS	*NRIP1*	hsa-miR-656	Low miRNA
MCFD2	*OSTM1*	hsa-miR-200c	High miRNA
CYB5B	*CREB1*	hsa-miR-577	Low miRNA
IQSEC3	*PTPRT*	hsa-miR-608	Low miRNA
CLDN1	*FGD6*	hsa-miR-186	Medium miRNA
GRIK5	*DGAT1*	hsa-miR-637	Low miRNA
CIAO1	*SELT*	hsa-miR-340	Low miRNA

KCTD3	*C14orf135*	hsa-miR-561	Low miRNA
FAM8A1	*AKAP11*	hsa-miR-519d	Medium miRNA
PAK6	*SLC7A8*	hsa-miR-185	Medium miRNA
TEAD1	*ARHGEF12*	hsa-miR-579	High miRNA
OTUB2	*PTPN3*	hsa-miR-16	High miRNA
FZD3	*GOLPH3L*	hsa-miR-539	Low miRNA
PRPF4B	*KRIT1*	hsa-miR-132	High miRNA
RNF138	*CPNE3*	hsa-miR-186	Medium miRNA
PRKCI	*KPNA1*	hsa-miR-577	Low miRNA
QKI	*SGPP1*	hsa-miR-656	Low miRNA
ACTR8	*LARP4*	hsa-miR-607	Low miRNA
TOMM70A	*FAM110B*	hsa-miR-607	Low miRNA
ENTPD7	*OTUB2*	hsa-miR-424	Low miRNA
CUL4B	*ANKFY1*	hsa-miR-561	Low miRNA
WNT4	*CACNA2D2*	hsa-miR-637	High miRNA
AFAP1	*PGPEP1*	hsa-miR-204	High miRNA
HELZ	*ZC3H11A*	hsa-miR-186	High miRNA
IQSEC3	*AAK1*	hsa-miR-637	Low miRNA
STEAP4	*OCLN*	hsa-miR-340	Medium miRNA
ICK	*C2orf3*	hsa-miR-142-5p	High miRNA
STX1A	*SNCB*	hsa-miR-663	Low miRNA
SCAMP4	*SSBP3*	hsa-miR-608	Low miRNA
SH3TC2	*SH3PXD2A*	hsa-miR-326	High miRNA
EREG	*FGF7*	hsa-miR-607	High miRNA
LASP1	*MTDH*	hsa-miR-150	Medium miRNA
PDXK	*TNS1*	hsa-miR-608	Low miRNA
SETD1B	*GOSR1*	hsa-miR-326	High miRNA
PNMA2	*TIAM1*	hsa-miR-583	Low miRNA
YWHAZ	*GPR107*	hsa-miR-544	Low miRNA
RHOQ	*LARP4*	hsa-miR-559	Low miRNA
KIAA0240	*MARK2*	hsa-miR-608	Low miRNA
RAB22A	*CBFA2T2*	hsa-miR-579	High miRNA
COL4A4	*OCLN*	hsa-miR-340	Medium miRNA
RBM8A	*GPR107*	hsa-miR-200c	Low miRNA
CBLL1	*MAP3K2*	hsa-miR-607	Low miRNA
SLC25A22	*TRIM3*	hsa-miR-608	Low miRNA

IRF4	*CCDC68*	hsa-miR-384	High miRNA
SLC12A3	*FSTL3*	hsa-miR-485-5p	Medium miRNA
B4GALT6	*ZNF238*	hsa-miR-587	Low miRNA
HIP1	*SLC35E1*	hsa-miR-520b	Low miRNA
CREB1	*FAM110B*	hsa-miR-607	Low miRNA
RAP2B	*SSR1*	hsa-miR-656	Low miRNA
SLC17A6	*SLC12A5*	hsa-miR-25	Low miRNA
EIF4E	*NDFIP1*	hsa-miR-105	Low miRNA
TCF4	*GJA1*	hsa-miR-186	Low miRNA
GCN1L1	*CHST3*	hsa-miR-607	Low miRNA
PTPRT	*AAK1*	hsa-miR-1	High miRNA
HELZ	*MECP2*	hsa-miR-544	Low miRNA
FTO	*MAP1B*	hsa-miR-583	Low miRNA
MYB	*PERP*	hsa-miR-150	High miRNA
UBE4B	*KPNA4*	hsa-miR-607	Low miRNA
DLGAP2	*CBX7*	hsa-miR-181a	High miRNA
NCDN	*KIAA1045*	hsa-miR-637	Low miRNA
APPBP2	*ACTR8*	hsa-miR-607	Low miRNA
SV2B	*SCN3B*	hsa-miR-485-5p	High miRNA
NCAM1	*APC*	hsa-miR-561	High miRNA
KIAA0513	*CNNM1*	hsa-miR-326	High miRNA
BMPR2	*ZC3H11A*	hsa-miR-186	High miRNA
PPM1H	*TMOD2*	hsa-miR-384	Low miRNA
PACS2	*SSBP3*	hsa-miR-608	Low miRNA
PIP4K2B	*ZER1*	hsa-miR-661	Low miRNA
RAB11A	*FBXW2*	hsa-miR-579	Low miRNA
DLGAP2	*KIAA0319*	hsa-miR-570	Low miRNA
SLC4A4	*SLC7A11*	hsa-miR-570	Low miRNA
PEX14	*S100PBP*	hsa-miR-650	High miRNA
TBL1XR1	*ZFHX3*	hsa-miR-570	Low miRNA
PPM1H	*ENTPD1*	hsa-miR-661	Low miRNA
NRIP3	*AAK1*	hsa-miR-485-5p	High miRNA
BICD2	*ZC3H11A*	hsa-miR-186	High miRNA
SSR1	*PDS5B*	hsa-miR-603	Low miRNA
LMO7	*OCLN*	hsa-miR-340	Medium miRNA
GPM6B	*POU3F2*	hsa-miR-579	High miRNA

RBM12	*PEX19*	hsa-miR-539	Low miRNA
ASTN1	*LSAMP*	hsa-miR-147	High miRNA
SPTBN4	*CRTC1*	hsa-miR-608	Low miRNA
ATXN1	*RCOR1*	hsa-miR-141	Low miRNA
MAP3K3	*ZER1*	hsa-miR-661	Low miRNA
CACNG2	*SLC8A2*	hsa-miR-608	Low miRNA
FZR1	*UBTF*	hsa-miR-608	Low miRNA
PAX8	*ATP11A*	hsa-miR-637	High miRNA
RBM8A	*ARIH2*	hsa-miR-200c	Low miRNA
ENAH	*PBRM1*	hsa-miR-607	Low miRNA
HELZ	*ZNF24*	hsa-miR-607	Low miRNA
EIF2C1	*ARHGAP5*	hsa-miR-607	Low miRNA
PCM1	*ETF1*	hsa-miR-150	Medium miRNA
LIMCH1	*BACE1*	hsa-miR-620	Low miRNA
APPL1	*LARP4*	hsa-miR-607	Low miRNA
PCSK2	*C18orf1*	hsa-miR-203	High miRNA
CLCN4	*AAK1*	hsa-miR-629	Low miRNA
SAR1B	*SMAD2*	hsa-miR-656	Low miRNA
LASP1	*CFLAR*	hsa-miR-603	Low miRNA
ZFHX3	*CYLD*	hsa-miR-579	Low miRNA
GABRA4	*DLGAP2*	hsa-miR-181c	Low miRNA
SYNJ2BP	*HEG1*	hsa-miR-520e	Low miRNA
TNFSF15	*SLC34A2*	hsa-miR-637	Low miRNA
PRKAA1	*RAB8B*	hsa-miR-559	Low miRNA
ARNT2	*PMP2*	hsa-miR-23b	Medium miRNA
ZBTB24	*LARP4*	hsa-miR-603	Low miRNA
ATXN1	*DNM3*	hsa-miR-203	High miRNA
TRIM29	*ATP2A3*	hsa-miR-214	Low miRNA
SMARCC2	*LARP1*	hsa-miR-150	Medium miRNA
KCTD2	*ATP2B4*	hsa-miR-608	Low miRNA
PTPRT	*PAIP2B*	hsa-miR-203	High miRNA
VGLL3	*OCLN*	hsa-miR-561	Medium miRNA
HEG1	*ATRN*	hsa-miR-570	Low miRNA
SH3TC2	*AAK1*	hsa-miR-20b	Low miRNA
IPO9	*PJA2*	hsa-miR-579	High miRNA
ELAVL2	*DLG2*	hsa-miR-485-3p	High miRNA

DUSP8	*BSN*	hsa-miR-647	Low miRNA
SNX19	*AKAP11*	hsa-miR-607	Low miRNA
BSN	*IQSEC3*	hsa-miR-149	High miRNA
ENTPD1	*ITGA4*	hsa-miR-579	Low miRNA
HIPK2	*TCF4*	hsa-miR-557	Low miRNA
CREB1	*SSR1*	hsa-miR-520g	Low miRNA
YWHAZ	*SACM1L*	hsa-miR-544	Low miRNA
SBF1	*TFE3*	hsa-miR-608	Low miRNA
PGR	*THSD4*	hsa-miR-570	High miRNA
RAB11FIP2	*RANBP2*	hsa-miR-607	Low miRNA
PPM1H	*AGPAT4*	hsa-miR-607	Low miRNA
IPO9	*MECP2*	hsa-miR-599	High miRNA
USP7	*EP400*	hsa-miR-603	Low miRNA
CREB1	*LPGAT1*	hsa-miR-607	Low miRNA
FOXO3	*ATRN*	hsa-miR-570	Low miRNA
ATRX	*PPP1R12A*	hsa-miR-607	Low miRNA
CACNG2	*SV2B*	hsa-miR-27b	Medium miRNA
EIF2C1	*GOSR1*	hsa-miR-608	Low miRNA
STXBP5L	*AAK1*	hsa-miR-580	Low miRNA
RANBP2	*LARP4*	hsa-miR-607	Low miRNA
SNX27	*PPP1R12A*	hsa-miR-607	Low miRNA
GABRA1	*SLC1A2*	hsa-miR-203	High miRNA
KCTD2	*SCAMP4*	hsa-miR-608	Low miRNA
ERLIN2	*PHACTR2*	hsa-miR-552	Low miRNA
QKI	*ARNT2*	hsa-miR-23a	Medium miRNA
ZNF652	*ITPR2*	hsa-miR-135a	Medium miRNA
CYB5B	*G3BP2*	hsa-miR-577	Low miRNA
ERLIN1	*ENTPD1*	hsa-miR-421	Medium miRNA
PCM1	*ANKRD12*	hsa-miR-150	Medium miRNA
PERP	*CORO2A*	hsa-miR-150	High miRNA
DLGAP2	*AAK1*	hsa-miR-488	High miRNA
TMCC1	*CAND1*	hsa-miR-607	Low miRNA
SH3TC2	*SH3PXD2A*	hsa-miR-608	Low miRNA
STX11	*FMO2*	hsa-miR-494	Low miRNA
ITCH	*TOLLIP*	hsa-miR-144	Low miRNA
SRGAP3	*AAK1*	hsa-miR-329	High miRNA

UBTF	*HCFC1*	hsa-miR-608	Low miRNA
SEC63	*ZNF148*	hsa-miR-186	Medium miRNA
ZEB1	*ATXN1*	hsa-miR-203	High miRNA
ATG9A	*CRTC1*	hsa-miR-637	Low miRNA
PGAP1	*S100PBP*	hsa-miR-583	Low miRNA
HIP1	*ASB1*	hsa-miR-302a	Low miRNA
ANK3	*AAK1*	hsa-miR-203	High miRNA
HIP1	*FBXL18*	hsa-miR-302d	Low miRNA
RGS1	*ITGAV*	hsa-miR-569	Low miRNA
KLC2	*POLH*	hsa-miR-661	Low miRNA
CRTC1	*PPP2R4*	hsa-miR-661	Low miRNA
FGF2	*CALML4*	hsa-miR-578	Low miRNA
TEAD1	*MAP1B*	hsa-miR-616	High miRNA
TRPS1	*TNRC6B*	hsa-miR-204	High miRNA
CDS2	*SELT*	hsa-miR-520g	Low miRNA
C5orf28	*TMEM33*	hsa-miR-369-3p	Medium miRNA
APPBP2	*SCAMP1*	hsa-miR-561	Low miRNA
CAB39	*METAP2*	hsa-miR-384	Low miRNA
MTMR9	*ICK*	hsa-miR-568	Medium miRNA
SLC12A5	*DLGAP2*	hsa-miR-92b	Low miRNA
ZNF592	*ARHGDIA*	hsa-miR-637	Low miRNA
POLI	*HLTF*	hsa-miR-410	Low miRNA
ZNF264	*ATRN*	hsa-miR-607	Low miRNA
SEMA5A	*TEAD1*	hsa-miR-616	High miRNA
GSPT1	*RBBP4*	hsa-miR-579	Low miRNA
QKI	*SMCR7L*	hsa-miR-200b	Low miRNA
FGF2	*MAP3K2*	hsa-miR-578	Low miRNA
SMARCD1	*PGPEP1*	hsa-miR-515-5p	Low miRNA
OCLN	*EHF*	hsa-miR-607	High miRNA
LBR	*ETV1*	hsa-miR-340	High miRNA
PCSK2	*RAB6B*	hsa-miR-329	High miRNA
THSD4	*MMP11*	hsa-miR-661	High miRNA
ZNF24	*PREPL*	hsa-miR-607	Low miRNA
KLHL2	*HLF*	hsa-miR-520g	Low miRNA
PLEKHB2	*UBE2D1*	hsa-miR-607	Low miRNA
GCN1L1	*TGOLN2*	hsa-miR-607	Low miRNA

ATP6V1C1	*MAP3K2*	hsa-miR-607	Low miRNA
PRPF4B	*FAM110B*	hsa-miR-607	Low miRNA
GRIN2A	*AAK1*	hsa-miR-136	High miRNA
APH1A	*CBX5*	hsa-miR-539	Low miRNA
IL6ST	*LSAMP*	hsa-miR-136	Medium miRNA
CELSR2	*MNT*	hsa-miR-608	Low miRNA
TRAF6	*ITGB8*	hsa-miR-607	High miRNA
PRDM2	*CEP68*	hsa-miR-211	High miRNA
SEC14L5	*ADARB2*	hsa-miR-326	High miRNA
BMPR2	*8-Sep*	hsa-miR-186	Medium miRNA
YWHAZ	*TNFAIP1*	hsa-miR-544	Low miRNA
SMAD3	*TNS1*	hsa-miR-587	Low miRNA
SH3TC2	*LDB3*	hsa-miR-150	Low miRNA
PCDH9	*PMP2*	hsa-miR-587	Medium miRNA
VPS26A	*ETF1*	hsa-miR-520h	Low miRNA
CLIC5	*ADAMTSL3*	hsa-miR-340	Medium miRNA
PIGA	*SORT1*	hsa-miR-587	Low miRNA
FOXN3	*RCOR1*	hsa-miR-141	High miRNA
CCDC68	*FGF7*	hsa-miR-340	Medium miRNA
TNPO1	*MIA3*	hsa-miR-186	Medium miRNA
PTPRT	*PCLO*	hsa-miR-203	High miRNA
UBE2J1	*LBR*	hsa-miR-607	Low miRNA
NOS1AP	*PAIP2B*	hsa-miR-329	High miRNA
GSPT2	*MAP1B*	hsa-miR-607	High miRNA
FAM8A1	*SMAD2*	hsa-miR-369-3p	Medium miRNA
TRPS1	*TSC1*	hsa-miR-204	Medium miRNA
RAB3B	*MCTP2*	hsa-miR-105	High miRNA
CCL22	*TRAF3*	hsa-miR-661	Medium miRNA
GOSR1	*LASP1*	hsa-miR-150	Medium miRNA
SV2B	*SLC1A2*	hsa-miR-203	High miRNA
TRPS1	*TMEM135*	hsa-miR-641	Low miRNA
C12orf5	*PLEKHB2*	hsa-miR-20a	Low miRNA
TRPS1	*TBL1X*	hsa-miR-539	Low miRNA
KCNS1	*AAK1*	hsa-miR-326	High miRNA
PIP4K2B	*PTPN18*	hsa-miR-661	Low miRNA
TEAD1	*CBX5*	hsa-miR-570	High miRNA

UBE4B	*LARP4*	hsa-miR-607	Low miRNA
ARL5A	*MCFD2*	hsa-miR-200b	High miRNA
ZNF652	*ARHGAP5*	hsa-miR-607	Low miRNA
CPT1A	*ATP8A1*	hsa-miR-579	High miRNA
TRPS1	*GOLPH3L*	hsa-miR-539	Low miRNA
GGCX	*KPNA1*	hsa-miR-577	Low miRNA
UBTF	*RAB3IL1*	hsa-miR-608	Low miRNA
SNPH	*SCN3B*	hsa-miR-485-5p	High miRNA
MYT1L	*RIMBP2*	hsa-miR-106a	Low miRNA
ZNF24	*SLC7A11*	hsa-miR-340	Low miRNA
TJP1	*KCTD3*	hsa-miR-561	Low miRNA
SGK3	*FBXL18*	hsa-miR-519d	Low miRNA
TRPS1	*VASH1*	hsa-miR-204	High miRNA
IQSEC3	*AAK1*	hsa-miR-625	High miRNA
PDLIM5	*ANKRD12*	hsa-miR-150	Medium miRNA
ZDHHC3	*DCP1A*	hsa-miR-544	Low miRNA
CBX5	*RHOBTB3*	hsa-miR-381	Low miRNA
POU4F2	*ONECUT2*	hsa-miR-23a	Low miRNA
MBNL3	*SLC7A2*	hsa-miR-200a	Medium miRNA
REEP5	*GPR107*	hsa-miR-587	Low miRNA
PPM1F	*ENTPD1*	hsa-miR-9	Medium miRNA
PRRG4	*PEX13*	hsa-miR-302a	High miRNA
EIF4EBP2	*PTPN18*	hsa-miR-34a	High miRNA
SDC3	*RBM15B*	hsa-miR-185	High miRNA
SEC23IP	*SNX27*	hsa-miR-607	Low miRNA
ZNF667	*KIF1C*	hsa-miR-520h	Low miRNA
KIAA0408	*ST8SIA3*	hsa-miR-522	Low miRNA
EPHA7	*GFRA1*	hsa-miR-539	Medium miRNA
HCFC1	*ATP6V0A1*	hsa-miR-608	Low miRNA
ZNF532	*RPS6KB1*	hsa-miR-200c	High miRNA
SNX27	*LPGAT1*	hsa-miR-607	Low miRNA
OCRL	*CLIP1*	hsa-miR-570	Low miRNA
C17orf63	*PHF21A*	hsa-miR-185	High miRNA
PEG3	*OSBPL2*	hsa-miR-607	Low miRNA
DLGAP2	*RAPGEFL1*	hsa-miR-424	Low miRNA
QKI	*EDNRB*	hsa-miR-607	High miRNA

ZNF264	*PHACTR2*	hsa-miR-603	Low miRNA
PPM1F	*TFE3*	hsa-miR-608	Low miRNA
HELZ	*PIP5K1C*	hsa-miR-608	Low miRNA
CALML4	*C18orf1*	hsa-miR-578	Low miRNA
POU2AF1	*CD96*	hsa-miR-485-5p	High miRNA
CEACAM5	*PTER*	hsa-miR-607	High miRNA
IQSEC3	*PRKAR1B*	hsa-miR-150	Medium miRNA
EIF4E	*GGCX*	hsa-miR-607	Low miRNA
FGF2	*ZNF654*	hsa-miR-578	Low miRNA
GPM6A	*WASF3*	hsa-miR-200c	Medium miRNA
PCSK2	*HTR2C*	hsa-miR-203	High miRNA
PREPL	*LPGAT1*	hsa-miR-607	Low miRNA
RAB3B	*ST8SIA3*	hsa-miR-203	Low miRNA
SLC6A8	*ARHGAP19*	hsa-miR-377	Medium miRNA
ARNT2	*TNPO1*	hsa-miR-23a	High miRNA
CYB5B	*HELZ*	hsa-miR-577	Low miRNA
GOSR1	*MAN2A2*	hsa-miR-326	Low miRNA
ICK	*FAM110B*	hsa-miR-607	Low miRNA
PTPRT	*CHD5*	hsa-miR-149	High miRNA
TBL1XR1	*ZEB2*	hsa-miR-367	Low miRNA
SMCHD1	*ATAD2*	hsa-miR-340	Low miRNA
NRIP1	*CAPRIN1*	hsa-miR-656	Low miRNA
RNF126	*RANBP3*	hsa-miR-637	Medium miRNA
SCAMP4	*TXLNA*	hsa-miR-608	Low miRNA
AMMECR1	*GFPT1*	hsa-miR-520h	Low miRNA
CCNH	*MRPS10*	hsa-miR-561	Low miRNA
TK2	*DOLPP1*	hsa-miR-326	Low miRNA
MAP3K2	*GAB1*	hsa-miR-20b	High miRNA
SBF1	*TRIM3*	hsa-miR-608	Low miRNA
PELI1	*PLEKHB2*	hsa-miR-607	Low miRNA
KIDINS220	*S100PBP*	hsa-miR-200a	High miRNA
HDAC2	*GTF2H5*	hsa-miR-495	Low miRNA
STEAP4	*CCRL1*	hsa-miR-340	Medium miRNA
ATP6V0A1	*SH3GLB2*	hsa-miR-608	Low miRNA
ELAVL1	*UBE2G1*	hsa-miR-539	Low miRNA
SPCS3	*UBE2D1*	hsa-miR-142-5p	High miRNA

CACNG2	*AAK1*	hsa-miR-204	Medium miRNA
TOMM70A	*ACTR8*	hsa-miR-607	Low miRNA
STAT3	*PPP3CA*	hsa-miR-495	Low miRNA
SEC16A	*HEG1*	hsa-miR-106b	Medium miRNA
SH3TC2	*ADARB2*	hsa-miR-661	Low miRNA
DSC3	*CHML*	hsa-miR-200b	Low miRNA
GIPC2	*CEACAM5*	hsa-miR-607	Low miRNA
GABRA1	*AAK1*	hsa-miR-580	Low miRNA
PPM1H	*ZNF592*	hsa-miR-661	Low miRNA
MBNL3	*G6PC*	hsa-miR-607	High miRNA
NCDN	*TRIM3*	hsa-miR-608	Low miRNA
AP2A2	*RAB11FIP3*	hsa-miR-661	Low miRNA
NDST3	*ONECUT2*	hsa-miR-579	Medium miRNA
CAMK2G	*NFIB*	hsa-miR-495	Low miRNA
SV2B	*RIMS3*	hsa-miR-7	High miRNA
SH3TC2	*ADARB2*	hsa-miR-599	Low miRNA
SLC7A8	*METTL7A*	hsa-miR-766	Low miRNA
CLDN8	*RAB3B*	hsa-miR-579	High miRNA
HELZ	*TMF1*	hsa-miR-607	Low miRNA
ENAH	*PTPN11*	hsa-miR-607	Low miRNA
RDX	*SSR1*	hsa-miR-603	Low miRNA
KIF3C	*FBXW2*	hsa-miR-24	High miRNA
RANBP10	*ARIH2*	hsa-miR-200c	Low miRNA
HBS1L	*PELI2*	hsa-miR-200c	High miRNA
ENTPD1	*BDKRB2*	hsa-miR-515-5p	Low miRNA
CCL22	*IL2RB*	hsa-miR-34a	Low miRNA
CUL4B	*PHKB*	hsa-miR-579	Low miRNA
GPR107	*ABI2*	hsa-miR-544	Low miRNA
CUL4A	*GFPT1*	hsa-miR-520g	Low miRNA
IPO9	*PURA*	hsa-miR-106b	Medium miRNA
AP1S2	*CTNND2*	hsa-miR-561	High miRNA
MINA	*ZNF148*	hsa-miR-577	Low miRNA
RBM12	*CPSF6*	hsa-miR-30d	Low miRNA
SNX27	*HECA*	hsa-miR-607	Low miRNA
UBE4B	*APPL1*	hsa-miR-607	Low miRNA
AAK1	*RIMS1*	hsa-miR-495	High miRNA

SNX27	*LARP4*	hsa-miR-607	Low miRNA
NOS1AP	*CAMKV*	hsa-miR-329	High miRNA
HELZ	*SMARCA5*	hsa-miR-561	Low miRNA
RAB11A	*DPP8*	hsa-miR-579	Low miRNA
AGPS	*GFPT1*	hsa-miR-520h	Low miRNA
KCNC1	*PTPRT*	hsa-miR-203	High miRNA
RBM15B	*PGPEP1*	hsa-miR-204	High miRNA
SLC17A6	*MYT1L*	hsa-miR-495	High miRNA
SGPP1	*MAP3K2*	hsa-miR-561	Low miRNA
TFE3	*C18orf1*	hsa-miR-608	Low miRNA
PDE4B	*NCOA1*	hsa-miR-369-3p	High miRNA
COL4A4	*ADAMTSL3*	hsa-miR-340	Medium miRNA
LRRC2	*PGPEP1*	hsa-miR-302a	Low miRNA
KCNC1	*MEF2C*	hsa-miR-203	High miRNA
P2RX1	*LAX1*	hsa-miR-147	High miRNA
KIF11	*GFPT1*	hsa-miR-520h	Low miRNA
CANX	*CPNE3*	hsa-miR-186	Medium miRNA
SV2B	*CAMKK2*	hsa-miR-7	High miRNA
TMEM106B	*RNF38*	hsa-miR-802	Low miRNA
ATP2B3	*GRIN2A*	hsa-miR-105	Low miRNA
TFE3	*RANBP3*	hsa-miR-608	Low miRNA
RBM15B	*NOTCH2*	hsa-miR-204	Medium miRNA
ICK	*PRPF4B*	hsa-miR-607	Low miRNA
BAHD1	*SLC23A2*	hsa-miR-326	Low miRNA
PDE4B	*PMP2*	hsa-miR-369-3p	High miRNA
BSN	*SLC17A7*	hsa-miR-185	High miRNA
FBXL7	*HGSNAT*	hsa-miR-570	Low miRNA
NLK	*AKAP11*	hsa-miR-607	High miRNA
RBM8A	*PRPF4B*	hsa-miR-606	Low miRNA
HCFC1	*MGRN1*	hsa-miR-608	Low miRNA
SLK	*PKD2*	hsa-miR-607	Low miRNA
GSPT1	*RAB1A*	hsa-miR-579	Low miRNA
SELT	*PFN2*	hsa-miR-520h	Low miRNA
AGPS	*DHTKD1*	hsa-miR-520h	Low miRNA
SSR1	*AKAP11*	hsa-miR-603	Low miRNA
C5orf22	*UBE2D1*	hsa-miR-142-5p	High miRNA

PRKCA	*SLC23A2*	hsa-miR-326	Low miRNA
SNX19	*FBXW2*	hsa-miR-607	Low miRNA
APPL1	*THRAP3*	hsa-miR-579	Low miRNA
TTC3	*CAND1*	hsa-miR-607	Low miRNA
CRNKL1	*TCF4*	hsa-miR-410	Low miRNA
RAB5A	*GOLPH3L*	hsa-miR-539	Low miRNA
NFYB	*LACTB2*	hsa-miR-559	Low miRNA
CYB5B	*KPNA1*	hsa-miR-577	Low miRNA
FOXN3	*TMEM57*	hsa-miR-141	High miRNA
TMCC1	*RBBP4*	hsa-miR-539	Low miRNA
SMC1A	*STX17*	hsa-miR-7	Low miRNA
KIF1B	*KIF3A*	hsa-miR-335	Low miRNA
MAP4K4	*USP25*	hsa-miR-559	Low miRNA
KIAA0513	*PARVA*	hsa-miR-326	Low miRNA
ZBTB6	*PIP4K2B*	hsa-miR-105	Low miRNA
ATP2B3	*KCNS1*	hsa-miR-326	High miRNA
CBFB	*CCDC93*	hsa-miR-559	Low miRNA
NCDN	*FBXO41*	hsa-miR-637	Low miRNA
MECP2	*PPP1R12B*	hsa-miR-558	Low miRNA
ENAH	*APPL1*	hsa-miR-607	Low miRNA
RAB6B	*CAMK2A*	hsa-miR-485-5p	High miRNA
OPA3	*PGPEP1*	hsa-miR-302a	Low miRNA
SLC7A11	*GJA1*	hsa-miR-607	Low miRNA
MECP2	*MGA*	hsa-miR-593	Low miRNA
PRPF4B	*TMF1*	hsa-miR-607	Low miRNA
CEP350	*ITGB8*	hsa-miR-19a	Low miRNA
BCLAF1	*PPP3CA*	hsa-miR-495	Low miRNA
THSD4	*KCNK15*	hsa-miR-15b	Low miRNA
TIGD6	*BMPR1B*	hsa-miR-607	Low miRNA
SLC17A6	*DLGAP2*	hsa-miR-363	Low miRNA
ELAVL1	*ARHGAP5*	hsa-miR-607	Low miRNA
POGK	*LPGAT1*	hsa-miR-607	Low miRNA
C6orf211	*SPG20*	hsa-miR-384	Low miRNA
SYP	*SLC25A22*	hsa-miR-608	Low miRNA
ZNF592	*APPL1*	hsa-miR-607	Low miRNA
PAX8	*LRRC15*	hsa-miR-326	High miRNA

CHML	*NPR3*	hsa-miR-340	Medium miRNA
FMO2	*SLCO4C1*	hsa-miR-340	Medium miRNA
TRPS1	*CNP*	hsa-miR-204	High miRNA
PAK2	*ZEB2*	hsa-miR-369-3p	Medium miRNA
TSPYL1	*GSPT1*	hsa-miR-485-5p	Low miRNA
TMED7	*RNF138*	hsa-miR-186	Medium miRNA
BSN	*SH3PXD2A*	hsa-miR-149	High miRNA
AAK1	*CACNB4*	hsa-miR-539	High miRNA
SLC7A2	*BRIP1*	hsa-miR-27a	High miRNA
GCNT2	*NPR3*	hsa-miR-539	Medium miRNA
HELZ	*TXLNA*	hsa-miR-646	Low miRNA
PTPN14	*PAPPA*	hsa-miR-195	Low miRNA
CCL22	*IL2RB*	hsa-miR-449b	Medium miRNA
SNX27	*RAB11FIP2*	hsa-miR-607	Low miRNA
KCNJ12	*CCDC134*	hsa-miR-125b	High miRNA
CLIC5	*FGF18*	hsa-miR-659	High miRNA
SNX27	*GCN1L1*	hsa-miR-607	Low miRNA
TRPS1	*HIPK2*	hsa-miR-204	High miRNA
TNKS2	*GSPT1*	hsa-miR-577	Low miRNA
HIP1	*OPA3*	hsa-miR-372	Low miRNA
BSN	*CNNM1*	hsa-miR-326	High miRNA
PACS2	*TRIM3*	hsa-miR-608	Low miRNA
EIF4EBP2	*MGAT1*	hsa-miR-31	High miRNA
SMG5	*SCAMP4*	hsa-miR-608	Low miRNA
VANGL1	*SLC7A8*	hsa-miR-185	Medium miRNA
PRNP	*MAP3K2*	hsa-miR-559	Low miRNA
KIAA0513	*ADARB2*	hsa-miR-326	High miRNA
KPNA4	*KIF1C*	hsa-miR-512-5p	Low miRNA
PAFAH2	*METTL8*	hsa-miR-520h	Low miRNA
AGPAT5	*CAPRIN1*	hsa-miR-656	Low miRNA
LIMS1	*CHST11*	hsa-miR-142-5p	High miRNA
MECP2	*FOXO3*	hsa-miR-593	Low miRNA
LANCL1	*C18orf1*	hsa-miR-204	Medium miRNA
NUBPL	*SERINC1*	hsa-miR-607	Low miRNA
MAP3K3	*HIF1AN*	hsa-miR-661	Low miRNA
MYNN	*C2orf3*	hsa-miR-607	Medium miRNA

DUSP8	*BSN*	hsa-miR-185	High miRNA
PIGK	*SERBP1*	hsa-miR-410	Low miRNA
TACC1	*LRPAP1*	hsa-miR-641	Low miRNA
KIAA0513	*AAK1*	hsa-miR-302a	Low miRNA
TGOLN2	*MTDH*	hsa-miR-607	Low miRNA
CDC42BPA	*SOBP*	hsa-miR-579	High miRNA
SMARCD1	*SSBP3*	hsa-miR-608	Low miRNA
RAB22A	*ZMIZ1*	hsa-miR-661	Low miRNA
PTGER2	*FGF7*	hsa-miR-607	High miRNA
BSN	*AAK1*	hsa-miR-647	Low miRNA
ENAH	*OPA1*	hsa-miR-607	Low miRNA
ZNF264	*SH3BP2*	hsa-miR-661	Low miRNA
AAK1	*CNTNAP2*	hsa-miR-302d	Low miRNA
PIP5K1C	*RAB11FIP3*	hsa-miR-661	Low miRNA
MCTS1	*YIPF6*	hsa-miR-410	Low miRNA
DSC3	*FGD6*	hsa-miR-656	Medium miRNA
UBTF	*ATP6V0A1*	hsa-miR-608	Low miRNA
ELAVL1	*PAICS*	hsa-miR-539	Low miRNA
TRAF3	*DSG3*	hsa-miR-29c	Low miRNA
SOSTDC1	*PRRG4*	hsa-miR-561	High miRNA
GNL3L	*CFLAR*	hsa-miR-552	Low miRNA
QKI	*APPL1*	hsa-miR-607	Low miRNA
KIAA0513	*AAK1*	hsa-miR-93	Low miRNA
NHLH2	*ONECUT2*	hsa-miR-340	Low miRNA
COL4A4	*VGLL3*	hsa-miR-23a	High miRNA
FBXO41	*KIAA0556*	hsa-miR-612	High miRNA
KPNA4	*TJP1*	hsa-miR-561	Low miRNA
ATP2B3	*AAK1*	hsa-miR-326	High miRNA
KLK10	*CDH1*	hsa-miR-612	Medium miRNA
DNAJC16	*LPGAT1*	hsa-miR-559	Low miRNA
CLCN4	*YTHDC1*	hsa-miR-607	Low miRNA
FBXO41	*FAIM2*	hsa-miR-185	High miRNA
MAPT	*HIPK2*	hsa-miR-623	Low miRNA
RAP2B	*ZMAT3*	hsa-miR-603	Low miRNA
PIP4K2B	*TJP1*	hsa-miR-105	Low miRNA
ATXN1	*HGSNAT*	hsa-miR-204	Medium miRNA

PTPRT	*AAK1*	hsa-miR-7	High miRNA
STX11	*CLIC5*	hsa-miR-539	Medium miRNA
TBC1D13	*PADI2*	hsa-miR-326	Low miRNA
DZIP3	*SLC7A11*	hsa-miR-494	High miRNA
RAD50	*TRPS1*	hsa-miR-539	Low miRNA
SNX27	*PTPN11*	hsa-miR-607	Low miRNA
SLC4A4	*ITIH5*	hsa-miR-186	Medium miRNA
CUL4B	*ABI2*	hsa-miR-579	Low miRNA
RAB14	*MTDH*	hsa-miR-607	Low miRNA
SYNGR3	*AAK1*	hsa-miR-185	High miRNA
KIAA0232	*TCF4*	hsa-miR-557	Low miRNA
RNF4	*MXI1*	hsa-miR-495	Low miRNA
SNX27	*RANBP2*	hsa-miR-607	Low miRNA
RTF1	*LARP4*	hsa-miR-603	Low miRNA
SH3TC2	*CLMN*	hsa-miR-429	Low miRNA
RBM8A	*JHDM1D*	hsa-miR-606	Low miRNA
GRIN2A	*AAK1*	hsa-miR-629	Low miRNA
SERBP1	*IMPACT*	hsa-miR-495	Low miRNA
ERO1LB	*NPR3*	hsa-miR-494	High miRNA
TJP1	*ASB1*	hsa-miR-105	Low miRNA
QKI	*SNX27*	hsa-miR-488	High miRNA
MXD4	*ACVR2B*	hsa-miR-138	High miRNA
TNPO1	*MID1*	hsa-miR-186	Medium miRNA
RAB5A	*PRPF40A*	hsa-miR-539	Low miRNA
CACNB4	*ENC1*	hsa-miR-539	High miRNA
NFAT5	*ARHGEF12*	hsa-miR-579	High miRNA
PRKACA	*TRIM3*	hsa-miR-608	Low miRNA
AGPAT5	*CTNND2*	hsa-miR-561	High miRNA
TRPS1	*NCOA3*	hsa-miR-577	Low miRNA
MRAS	*SYNJ1*	hsa-miR-152	High miRNA
ZNF532	*RPS6KB1*	hsa-miR-200b	High miRNA
DYNC1LI2	*TSC1*	hsa-miR-130a	Medium miRNA
CDC73	*MCFD2*	hsa-miR-429	Low miRNA
SSR1	*TMEM30A*	hsa-miR-587	Low miRNA
ZFP37	*UCHL5*	hsa-miR-607	Low miRNA
DLGAP2	*FBXW7*	hsa-miR-92b	Low miRNA

SLC25A4	*AGPAT5*	hsa-miR-552	High miRNA
