# Supplementary material for: Differential correlation analysis of glioblastoma reveals immune ceRNA interactions predictive of patient survival
Source: BMC Bioinformatics. 2017 Feb 28;18:132. doi: 10.1186/s12859-017-1557-4 (PMC5330036; doi:10.1186/s12859-017-1557-4)
Supplement: Additional file 4: Table S2. — Top overrepresented ceRNAs in the core ceRNA network. (PDF 67 kb) [file 12859_2017_1557_MOESM4_ESM.pdf]

Table S2. Top overrepresented ceRNAs in the core ceRNA network

ceRNA	Degree (percentage*)	*P*-value**
AAK1	89 (5.05%)	1.61E-47
BSN	34 (1.93%)	1.10E-32
SV2B	43 (2.44%)	8.05E-31
SSBP3	18 (1.02%)	2.43E-29
YWHAZ	13 (0.74%)	2.09E-24
SNX27	28 (1.59%)	2.31E-23
SLC8A2	20 (1.14%)	2.35E-22
KIAA1045	21 (1.19%)	2.18E-20
OSBPL2	16 (0.91%)	1.03E-19
NCDN	16 (0.91%)	7.75E-19

*Percentages in the core network.

**One-tailed Fisher's exact test *P*-value for overrepresentation.
