# Supplementary material for: Differential correlation analysis of glioblastoma reveals immune ceRNA interactions predictive of patient survival
Source: BMC Bioinformatics. 2017 Feb 28;18:132. doi: 10.1186/s12859-017-1557-4 (PMC5330036; doi:10.1186/s12859-017-1557-4)
Supplement: Additional file 6: Table S3. — Top functional clusters of core ceRNAs. (PDF 2280 kb) [file 12859_2017_1557_MOESM6_ESM.pdf]

Table S3. Top functional clusters of core ceRNAs

Category	Term	Num. ceRNAs	Fisher's exact *P*
**Cluster 1 (DAVID enrichment score = 6.13)**			
GOTERM_CC_FAT	GO:0044459: plasma membrane part	206	1.47E-12
GOTERM_CC_FAT	GO:0005887: integral to plasma membrane	99	3.64E-04
GOTERM_CC_FAT	GO:0031226: intrinsic to plasma membrane	99	7.69E-04
**Cluster 2 (DAVID enrichment score = 5.49)**			
SP_PIR_KEYWORDS	cell junction	51	1.31E-08
GOTERM_CC_FAT	GO:0030054: cell junction	59	1.75E-06
GOTERM_CC_FAT	GO:0045202: synapse	43	1.29E-05
SP_PIR_KEYWORDS	synapse	28	2.30E-05
GOTERM_CC_FAT	GO:0044456: synapse part	32	5.26E-05
**Cluster 3 (DAVID enrichment score = 5.18)**			
GOTERM_CC_FAT	GO:0031982: vesicle	73	4.61E-07
GOTERM_CC_FAT	GO:0031410: cytoplasmic vesicle	69	1.62E-06
GOTERM_CC_FAT	GO:0016023: cytoplasmic membrane-bounded vesicle	58	2.23E-05
SP_PIR_KEYWORDS	cytoplasmic vesicle	30	2.47E-05
GOTERM_CC_FAT	GO:0031988: membrane-bounded vesicle	59	2.91E-05
**Cluster 4 (DAVID enrichment score = 5.18)**			
SP_PIR_KEYWORDS	protein transport	60	1.98E-09
GOTERM_BP_FAT	GO:0045184: establishment of protein localization	82	1.17E-06
GOTERM_BP_FAT	GO:0015031: protein transport	81	1.60E-06
GOTERM_BP_FAT	GO:0008104: protein localization	89	3.93E-06
GOTERM_BP_FAT	GO:0046907: intracellular transport	70	8.36E-06
GOTERM_BP_FAT	GO:0006886: intracellular protein transport	43	1.20E-04
GOTERM_BP_FAT	GO:0034613: cellular protein localization	44	4.63E-04
GOTERM_BP_FAT	GO:0070727: cellular macromolecule	44	5.39E-04

localization			
**Cluster 5 (DAVID enrichment score = 4.03)**			
GOTERM_BP_FAT	GO:0019226: transmission of nerve impulse	46	2.13E-06
GOTERM_BP_FAT	GO:0007267: cell-cell signaling	66	5.69E-06
GOTERM_BP_FAT	GO:0007268: synaptic transmission	39	1.58E-05
GOTERM_BP_FAT	GO:0050877: neurological system process	78	0.41
