# Supplementary material for: Differential correlation analysis of glioblastoma reveals immune ceRNA interactions predictive of patient survival
Source: BMC Bioinformatics. 2017 Feb 28;18:132. doi: 10.1186/s12859-017-1557-4 (PMC5330036; doi:10.1186/s12859-017-1557-4)
Supplement: Additional file 8: Table S4. — Prognostic ceRNA triplets. (PDF 925 kb) [file 12859_2017_1557_MOESM8_ESM.pdf]

Table S4. Prognostic ceRNA triplets

ceRNA1	ceRNA2	miRNA	Log-rank *P*[*]	Comparison
IRF4	*CCL22*	hsa-miR-34a	1.92E-07	Low miRNA vs. others
IRF4	*IL2RB*	hsa-miR-34a	1.92E-07	Low miRNA vs. others
CCL22	*IL2RB*	hsa-miR-34a	1.92E-07	Low miRNA vs. others
IRF4	*CLDN18*	hsa-miR-34a	2.01E-05	Medium miRNA vs. others
MDM4	*PLEKHA6*	hsa-miR-622	5.32E-04	Medium miRNA vs. others

[*]ceRNA triplets with log-rank P<1E-3
